# Psychedelic perceptions: mental health service user attitudes to psilocybin therapy

**DOI:** 10.1007/s11845-021-02668-2

**Published:** 2021-06-15

**Authors:** Kate Corrigan, Maeve Haran, Conor McCandliss, Roisin McManus, Shannon Cleary, Rebecca Trant, Yazeed Kelly, Kathryn Ledden, Gavin Rush, Veronica O’Keane, John R. Kelly

**Affiliations:** 1 Tallaght University Hospital, Dublin, Ireland; 2 St. Patrick’s University Hospital, Dublin, Ireland; 3 School of Biomedical & Biomolecular Science, University College Dublin, Dublin, Ireland; 4 Royal College of Surgeons in Ireland, Dublin, Ireland; 5 Department of Psychiatry, Trinity College Dublin, Dublin, Ireland

**Keywords:** Attitudes, Hallucinogens, Lysergic acid diethylamide, Psilocybin, Psilocybin therapy, Psychedelics, Psychiatry

## Abstract

**Introduction:**

Despite the rapid advance of psychedelic science and possible translation of psychedelic therapy into the psychiatric clinic, very little is known about mental health service user attitudes.

**Objectives:**

To explore mental health service user attitudes to psychedelics and psilocybin therapy.

**Methods:**

A questionnaire capturing demographics, diagnoses, previous psychedelic and other drug use, and attitudes to psychedelics and psilocybin therapy was distributed to mental health service users.

**Results:**

Ninety-nine participants completed the survey (52% female, mean age 42 years). The majority (72%) supported further research, with 59% supporting psilocybin as a medical treatment. A total of 27% previously used recreational psilocybin, with a male preponderance (p = 0.01). Younger age groups, those with previous psychedelic experience, and those with non-religious beliefs were more likely to have favourable attitudes towards psilocybin. A total of 55% of the total sample would accept as a treatment if doctor recommended, whereas 20% would not. Fewer people with depression/anxiety had used recreational psychedelics (p = 0.03) but were more likely to support government funded studies (p = 0.02). A minority (5%) of people with conditions (psychosis and bipolar disorder) that could be exacerbated by psilocybin thought it would be useful for them. One fifth of the total sample viewed psychedelics as addictive and unsafe even under medical supervision. Concerns included fear of adverse effects, lack of knowledge, insufficient research, illegality, and relapse if medications were discontinued.

**Conclusions:**

The majority supported further research into psilocybin therapy. Younger people, those with previous recreational psychedelic experience, and those with non-religious beliefs were more likely to have favourable attitudes towards psilocybin therapy.

**Supplementary information:**

The online version contains supplementary material available at 10.1007/s11845-021-02668-2.

## Introduction

Translational psychedelic science is evolving rapidly [1, 2]. Preliminary clinical evidence suggests that the synergistic combination of psychedelic compounds with psychological support may improve outcomes in major depressive disorder (MDD) [3, 4], treatment-resistant depression (TRD) [5, 6], and addiction disorders [7–10]. Exploratory studies suggest potential benefits of psilocybin therapy in OCD [11], eating disorders [12], and migraine suppression [13], with ongoing clinical trials of psychedelic therapy in MDD, TRD, bipolar disorder type II depression, anxiety, alcohol use disorder, smoking cessation, cocaine addiction, anorexia nervosa, depression with mild cognitive impairment, OCD, and various types of headaches [14]. Results from ongoing well-powered double-blind randomized controlled trials (RCTs) will determine whether psychedelic therapy translates into clinical benefits for non-psychotic disorders in clinical psychiatry. More broadly, it has been proposed that psychedelic therapy may attenuate restricted and maladaptive habitual patterns of cognition and behaviour to facilitate the adoption of more healthy behavioural patterns [15].

The neurobiological mechanisms underlying psychedelic therapy are multi-modal, crossing molecular, cellular, and network systems. The primary initial pharmacological target appears to be activation of 5-HT2A receptors particularly in cortical layer 5 pyramidal cells [16–25], though 5-HT2A independent mechanisms may also be important [26]. Psychedelics promote structural and functional neural plasticity [27–31] and both preclinical [29] and clinical neuroimaging data suggests that psychedelics lead to 5-HT2A receptor-mediated glutamate release in the medial prefrontal cortex [32]. Psychedelics induce changes in global brain connectivity [33, 34], including default mode network (DMN) integrity [35] and amygdala reactivity [36, 37].

However, psychedelic compounds induce a wide range of complex subjective experiences with marked individual variation, even in the therapeutic setting and are contraindicated in psychosis spectrum disorders and mania [38, 39]. Furthermore, caution is required for those with a family history of psychosis. The individual variation and divergence between potential therapeutic utility for some disorders and contraindication in others demands meticulous, high-quality research that incorporates a precise-personalized-psychedelic therapy paradigm [40, 41]

There are marked differences between recreational and therapeutic uses of psychedelics [42–44]. Psilocybin therapy data from John Hopkins University, over a 16-year period, encompassing 250 volunteers and 380 sessions, reported no major psychological issues, with 0.9% of volunteers experiencing minor and transient psychological issues [42]. This contrasts with a survey of 1993 recreational psilocybin users, of which 7.6% reported seeking treatment for psychological symptoms they attributed to their challenging psilocybin experience [42]. This included three self-reported cases of incipient and enduring psychotic symptoms (at least one year) and three cases of attempted suicide [42].

In parallel to the “Psychedelic Revolution in Psychiatry” [45], recreational use appears to be increasing [46–51]. A recent survey conducted from March to May 2020 by the European Monitoring Centre for Drugs and Drug Addiction with 10,000 respondents from across Europe (average age 29 years, 58% male) showed that 10% used lysergic acid diethylamide (LSD) in the last 30 days [52]. Nationally representative data from the Crime Survey for England and Wales (2018/2019) reported that 0.5% of 16–59 years used magic mushrooms in the last 12 months, whereas in young adults (16–24 years), the rate was 1.6% [53]. A decade ago, Irish data from a representative number of people aged between 15 and 64 years (n = 7669 respondents) indicated that 7% had previously used magic mushrooms in their lifetime (6.5% of men and 4% of women) and 0.5% had used magic mushrooms in the previous year [54]. A recent survey of American university students (n = 3525) showed that 11% had previously used psychedelics [55].

Notwithstanding the self-selection bias inherent in the Global Drugs Surveys (GDS), the most recent GDS of 26,000 people, of whom 72% were males and approximately 1% were Irish, revealed some interesting trends [49]. This survey demonstrated increasing psychedelic use and reported that 55% of respondents used magic mushrooms in the last 12 months, mostly for “well-being,” but also for “self-medication” [49]. Worryingly, 40% of those who reported using psychedelics did not undergo any preparatory or integration sessions [49]. In addition, almost a quarter of respondents self-reported psychedelic microdosing over the last 12 months [49]. Furthermore, 4.2% of those using recreational psychedelics for emotional distress or psychiatric conditions sought emergency medical treatment over the last 12 months, including people with self-reported psychotic (0.2%) and bipolar (2.1%) disorders [49].

In terms of acceptability of psychedelic therapy, 35% of American college students (n = 124) agreed that psychedelics can be a therapeutic tool for those with depression and 39% for anxiety [56], with a much higher proportion (84%) supporting further research [56]. Interestingly, these attitudes were approximately in line with an online survey of American psychiatrists (n = 324), in which 42% reported that psychedelics show promise in treating some psychiatric disorders, with a quarter thinking psychedelics are unsafe even under medical supervision [57]. Echoing the college students, approximately 80% agreed that psychedelics deserve further research, with males and early career stage trainees (< 40 years) having more favourable attitudes towards the therapeutic use of psychedelics [57].

Results from the previous GDS (2019) of 85,000 people showed that 59% of people who previously used psychedelics said they would accept it as treatment for depression or PTSD, compared to only 18% of those surveyed who had never used psychedelics [58]. Of the respondents who would not accept PT, concerns related to “brain damage and bad trips” [58].

Society’s relationship and attitude towards psychedelics are complex, but if we are to consider using these compounds in clinical practice, it is essential to look beyond past mistakes of over-exuberance and non-scientific ideology, towards a shared transparent scientific understanding of the risks and benefits of psychedelic therapy [59]. This is all the more important in the context of increasing rates of recreational psychedelic use, the potential of psychedelics to exacerbate underlying pre-dispositions to psychosis and mania, together with psilocybin therapy being on the verge of translating into the psychiatric clinic for a range of non-psychotic psychiatric disorders [4].

Clear communication between researchers, clinicians, service users, and the public is required for the shared scientific understanding of the risks and potential benefits of psychedelic therapy and is a vital component in “keeping the Renaissance From Going Off the Rails” [60, 61]. This study will investigate mental health service user attitudes to psychedelics and psilocybin therapy.

## Methods

## Ethical approval

Tallaght University Hospital/St. James’s Hospital Joint Research Ethics Committee and St. Patrick’s University Hospital (SPUH) approved this study.

### Survey design

A questionnaire was designed based on previous studies [56, 57] to investigate the attitudes of mental health service users to psychedelics and psilocybin with psychological support (psilocybin therapy). Demographic details (including level of education, employment status, and religion), mental health diagnosis, and personal history of recreational drug use were recorded. A 5-point Likert scale (strongly agree, agree, do not know/neutral, disagree, and strongly disagree) was used to capture attitudes about psychedelics and psilocybin therapy. The last question (*I would be willing to gradually come off my medications in order to accept psilocybin (magic mushrooms) with psychological support if a doctor recommended it*) contained a free text option (*If you would not accept it, why not?*). See supplementary information (SI) for questionnaire.

### Participant recruitment

Participants were recruited from both Tallaght Community Mental Health Service and SPUH. Participants were eligible for the study if they were over 18 years of age and spoke fluent English. Recruitment from Tallaght Community Mental Health Service occurred through outpatient department (OPD) clinics from September to December 2020. Individuals were asked whether they would like to participate during routine OPD appointments by researchers (K.C., CMcC, S.C., and R.T.). As most of the clinics were via telephone due to the COVID-19 pandemic, once verbal consent was obtained, the participant pack was sent via post to their residence. The participant pack contained a cover sheet, consent form, Patient Information Leaflet (PIL), and the study questionnaire. Participants were contacted via telephone and prompted to return the questionnaire via post or email or were offered the opportunity to complete the questionnaire via telephone.

In SPUH, participants were recruited from inpatients on open wards from October to December 2020. Researchers (M.H. and R.McM) approached eligible patients, explained the study, provided the PIL, consent form, study questionnaire, and collected completed forms either later that day or on a subsequent day if a participant required more time. Inpatients on the acute locked ward were excluded due to concerns that participation may exacerbate distress or confusion in an acutely unwell population group.

### Data analysis

The number of people who responded Agree strongly and Agree were summed and presented as net agree percentages. Two-tailed chi-square tests in SPSS 26 were used to determine statistical significance (p ≤ 0.05) of the net Agree versus net Disagree proportions. See supplementary information for raw data. GraphPad was used for the figures. IBM SPSS Text Analytics for Surveys V 4.0.1 was used to analyse the free text.

### Diagnosis

Diagnoses were compressed into 5 groups to aid analysis (depression/anxiety, Bipolar affective disorder, psychotic disorders, personality disorders, and addiction disorders). There was only one respondent with a diagnosis of eating disorder, and they were excluded from the diagnosis analysis.

### Possible indication vs contra-indication

Psychotic disorders and bipolar affective disorder (BPAD) were included in the contra-indication group. The depression/anxiety and addiction groups were included in the possible indication group. People with Personality disorders were excluded.

## Results

Ninety-nine participants completed the survey, of which 56 were from the Tallaght community mental health service and 43 were inpatients in SPUH. A total of 105 mental health service users in Tallaght were asked if they wanted to participate. Two people declined and two later withdrew consent to participate. Of the total that initially agreed to take part, 56 participants returned the questionnaire (response rate of 53%). In the SPUH inpatient cohort, three people declined to take part (response rate of 95%).

### Demographics and diagnoses

See Table 1 for demographic characteristics and diagnoses.

**Table 1:** Demographic characteristics and diagnoses

Total (n = 99)	
Age (mean (SD), range)	41.68 (13.98), 19–73
Sex (% female)	51.5
Nationality (% Irish)	92.6
Education (%)	
< 16	13.1
Junior cert	15.2
Leaving cert	26.3
Some university	16.2
Bachelors	19.2
Postgrad	10.1
Employment (%)	
Student	6.1
Unemployed	31.3
Part time	10.1
Full time	38.4
Retired	14.1
Religion (%)	
None	28.6
Christian	66.3
Other	5.1
Diagnosis (%)	
Depression/Anxiety	36.4
Bipolar disorder	12.1
Psychotic disorders	17.2
Personality disorders	14.1
Addiction	19.2
Eating disorders	1.0

### Total sample attitudes

#### Attitudes to psilocybin therapy for various conditions

**Fig. 1:**
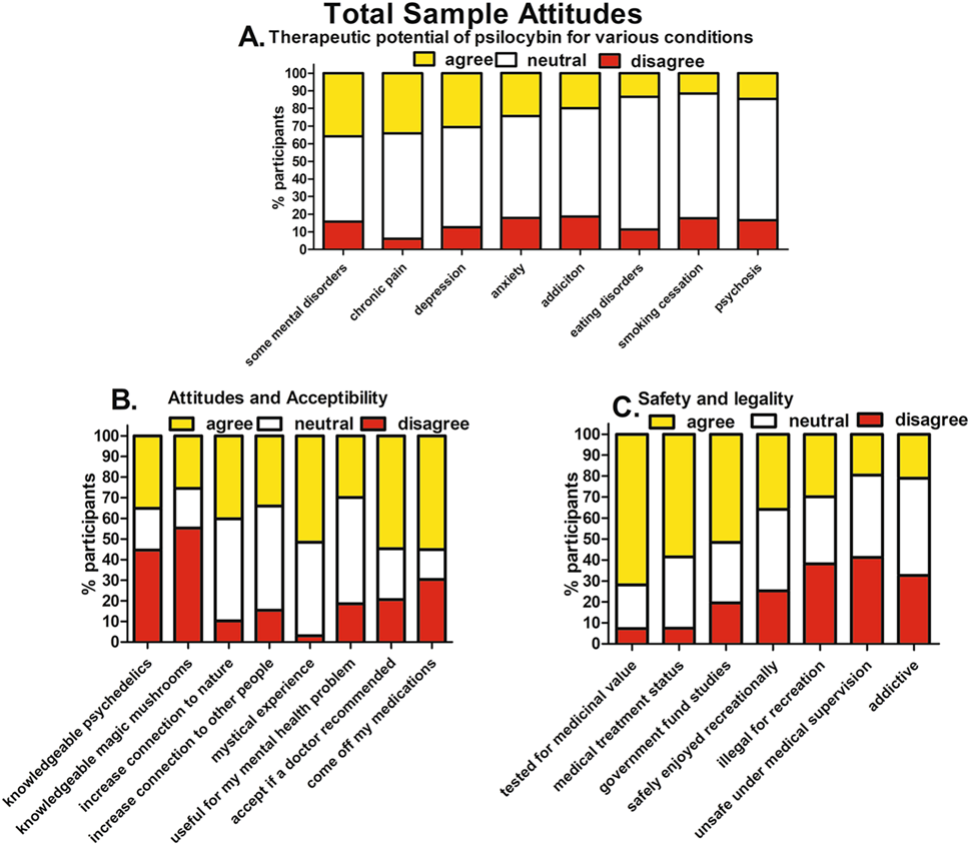
Total Sample attitudes to psilocybin therapy. **A** *Psilocybin therapy for various conditions*; 36% agreed that psilocybin could be useful for some mental disorders, 34% for chronic pain, 31% for depression, 24% for anxiety, 20% for addiction disorders, 15% for psychotic disorders, 13% for eating disorders, and 11% for smoking cessation. **B** *Attitudes and acceptability*: 35% reported being knowledgeable about psychedelic drugs and 26% about magic mushrooms (psilocybin). Exactly 40% agreed that psilocybin could increase connection to nature, 34% increase connection to other people, and 52% could lead to a mystical experience. A total of 30% agreed that psilocybin would be useful for their own mental health problem, 55% would accept psilocybin therapy if a doctor recommended it, and 55% would be willing to come off their medications to avail of psilocybin therapy. **C** *Attitudes to safety and legality*: 72% agreed that psilocybin should be tested for medicinal value and 59% believed that psilocybin should be granted medical treatment status; 52% thought that the government should fund psilocybin studies. Exactly 36% agreed that psilocybin could be safely enjoyed recreationally, whereas 30% agreed that it should remain illegal for recreational purposes. A total of 21% thought that psychedelics are addictive and 20% agreed that psychedelics are unsafe even under medical supervision

Of all participants, 36% agreed that psilocybin could be useful for some mental disorders, 34% for chronic pain, 31% for depression, 24% for anxiety, 20% for addiction disorders, 15% for psychotic disorders, 13% for eating disorders, and 11% for smoking cessation (Fig. 1A; Table S1).

### Attitudes and acceptability

35% of the total sample reported being knowledgeable about psychedelics and 26% about magic mushrooms (psilocybin). A total of 40% agreed that psilocybin could increase connection to nature, 34% connection to other people, and 52% could lead to a mystical experience. Exactly 30% agreed that psilocybin would be useful for their mental health problem, 55% would accept psilocybin therapy if a doctor recommended it, and 55% would be willing to come off their current medications to avail of psilocybin therapy (Fig. 1B; Table S1).

### Attitudes to safety and legality

A total of 72% agreed that psilocybin should be tested for medicinal value, and 59% believed that psilocybin should be granted medical treatment status. 52% thought that the government should fund psilocybin studies. Thirty-six percent agreed that psilocybin could be safely enjoyed recreationally, whereas 30% agreed that it should remain illegal for recreational purposes. A total of 21% believed that psychedelics are addictive and 20% that psychedelics are unsafe even under medical supervision (Fig. 1C; Table S1).

### Previous drug use

**Fig. 2:**
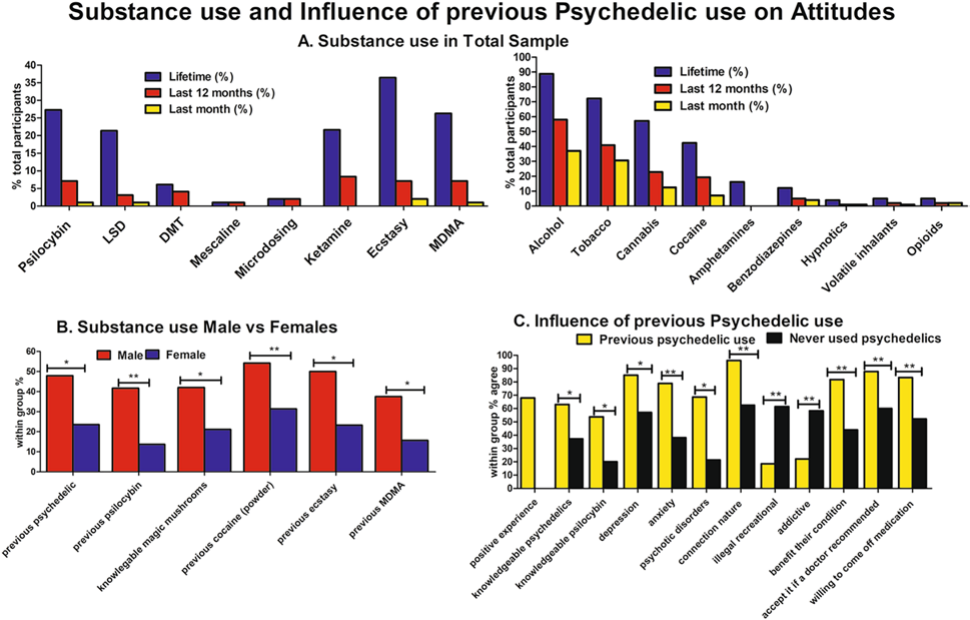
Previous psychedelic use associated with more favourable attitudes to psilocybin therapy. **A** Thirty-five percent of the total sample self-reported lifetime psychedelic use. Exactly 27% previously used magic mushroom (psilocybin), 7.1% in the last 12 months, and 1% in last month. **B** Males self-reported higher lifetime use of psychedelics (p = 0.01) and magic mushrooms (p = 0.002) compared to females. Males also reported higher lifetime use of powder cocaine use (p = 0.02), ecstasy (p = 0.006), and MDMA (p = 0.01). Males reported being more knowledgeable about magic mushrooms (p = 0.048). **C** Compared to those who have never used psychedelics, participants that had previously used psychedelics were more likely to self-report being knowledgeable about psychedelics (p = 0.01) and magic mushrooms (p = 0.003) agree that psilocybin could be a therapeutic tool for depression (p = 0.050), anxiety (p = 0.009), and psychotic disorders (p = 0.01) and would benefit their own mental health condition (p = 0.008). They were more like to accept psilocybin if a doctor recommended it (p = 0.008) and to come off medications (p = 0.006). They were less likely to think that recreational psilocybin should be illegal (p = 0.001) and more likely to agree that psilocybin can increase people’s connection to nature (p = 0.004)

 35% of the total sample self-reported lifetime psychedelic use. A total of 27% previously used magic mushrooms (psilocybin), 7% in the last 12 months, and 1% in last month. Twenty-one percent reported lifetime LSD use, 3% in last 12 months, and 1% in the last month. Six percent reported lifetime use of DMT and 4% last 12 months. One percent reported lifetime mescaline use, with the 1% using it in last 12 months. Two percent self-reported psychedelic microdosing (1 depression/anxiety, 1 addiction) in last 12 months, and 2% had previously attended a psychedelic retreat (1 depression/anxiety, 1 BPAD). See Fig. 2a and Table S2 for percent of all substances. Of those that had previously used psychedelics, 68% reported that it was a positive experience.

### Influence of previous psychedelic use

Participants that had previously used psychedelics were more likely to self-report being knowledgeable about psychedelics (p = 0.01) and magic mushrooms (p = 0.003) compared to those who have never used psychedelics. They were more likely to agree that psilocybin could be a therapeutic tool for depression (p = 0.050), anxiety (p = 0.009), psychotic disorders (p = 0.01), and would benefit their own mental health condition (p = 0.008). Similarly, they were more likely to accept psilocybin if a doctor recommended it (p = 0.008) and to come off medications (p = 0.006). They were less likely to think that recreational psilocybin should be illegal (p = 0.001) and more likely to agree that psilocybin can increase people’s connection to nature (p = 0.004) compared to those who have never used psychedelics (Fig. 2A; Table S3, S4).

### Influence of gender

Males self-reported significantly higher lifetime use of psychedelics (p = 0.01) and magic mushrooms (p = 0.002) and reported being more knowledgeable about magic mushrooms (p = 0.048) compared to females. Males also reported higher lifetime use of powder cocaine (p = 0.02), ecstasy (p = 0.006), and MDMA (p = 0.01). There were no other significant differences between males and females (Fig. 2B; Table S5).

### Influence of age

**Fig. 3:**
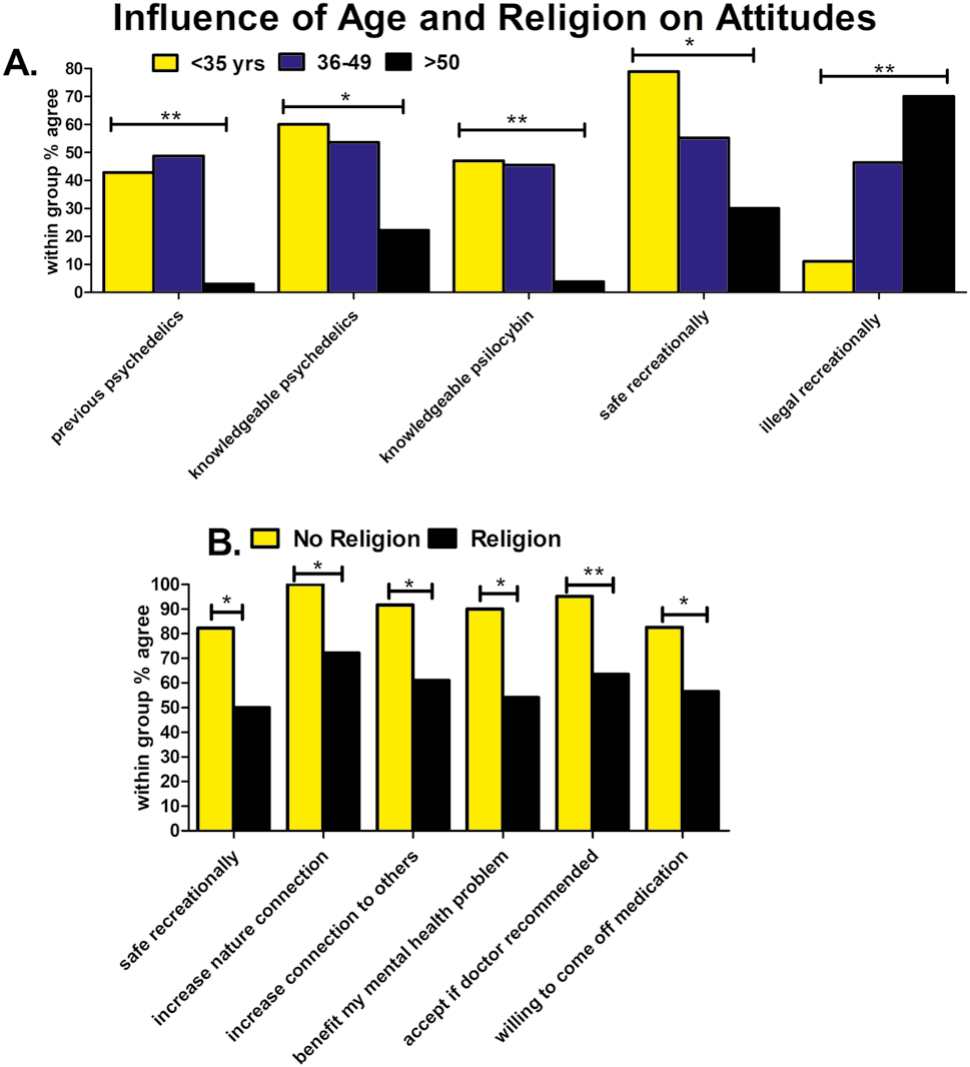
Younger age and non-religious beliefs associated with more favourable attitudes to psilocybin therapy. A Younger age groups reported higher levels of previous psychedelic use (p = 0.002), more knowledge about psychedelics (p = 0.016), and magic mushrooms (p = 0.001) were more likely to view psilocybin safe for recreational use (p = 0.03), and the youngest age group was less likely to agreed that psilocybin should be illegal (p = 0.001). **B** Participants with no religious beliefs were more likely to agree that psilocybin is safe recreationally (p = 0.02), increase nature connection (p = 0.033), increase connection to others (p = 0.048), benefit my mental health problem (p = 0.038), accept if doctor recommended (p = 0.006), and willing to come off medication (p = 0.03) compared to those with religious beliefs

The younger age groups reported higher levels of previous psychedelic use (p = 0.002) and more knowledge about psychedelics (p = 0.016) and magic mushrooms (p = 0.001). Younger age groups were also more likely to view psilocybin safe for recreational use (p = 0.034), and the youngest age group was less likely to agreed that psilocybin should be illegal (p = 0.001) (Fig. 3; Table S6).

### Influence of religion

Participants that self-reported no religious beliefs were more likely to agree that psilocybin is safe recreationally (p = 0.02), could increase nature connection (p = 0.03), increase connection to others (p = 0.048), benefit my mental health problem (p = 0.038), would accept if doctor recommended (p = 0.006), and would be willing to come off medication (p = 0.03) compared to those with religious beliefs (Fig. 3; Table S7).

### Influence of diagnosis

**Fig. 4:**
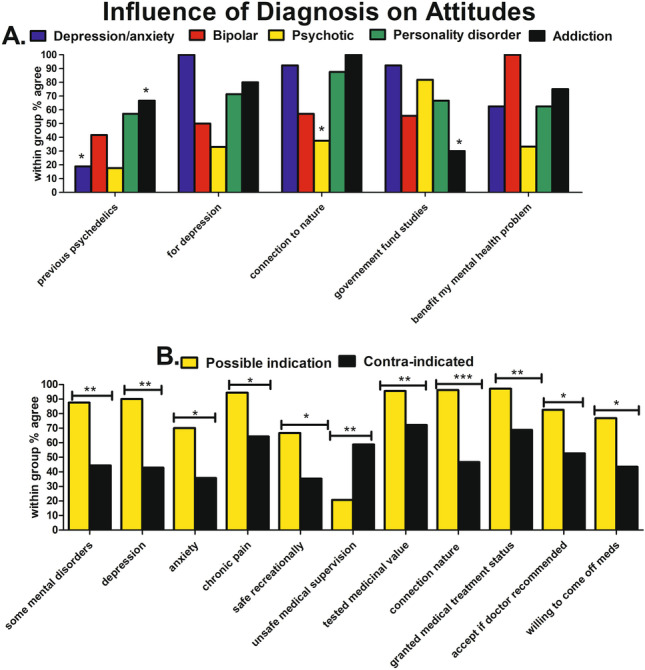
Influence of diagnosis on attitudes to psilocybin therapy. **A** Fewer people with depression/anxiety previously used psychedelics (*p* = 0.03) yet were more likely to agree that the government should fund studies (*p* = 0.02). More people with addiction disorders previously used psychedelics (p = 0.01), and this group was less likely to agree that the government should fund studies (p = 0.01). Participants with psychotic disorders disagreed that psychedelics increase connection to nature (p = 0.01). **B** Diagnoses with a possible therapeutic indication were more likely to agree that psilocybin may be useful for some mental health disorders (p = 0.003), for depression (p = 0.003), anxiety (p = 0.048), chronic pain (p = 0.03), safe recreationally (p = 0.03), increases connection to nature (p < 0.001), should be tested for medicinal value (p = 0.009), granted medical treatment status (p = 0.004), would accept if doctor recommended (p = 0.016), and willing to come off medications (p = 0.08). Those with diagnoses with a possible therapeutic indication were more likely to disagree that psychedelics are unsafe even under medical supervision (p = 0.009)

There were significant differences across the diagnostic groups in previous psychedelic use (p = 0.006), psilocybin for depression (p = 0.036), psilocybin increases connection to nature (p = 0.003), psilocybin should be tested for medicinal value (p = 0.031), and government should fund psilocybin studies (p = 0.006) (Fig. 4; Table S8, S9). Post hoc analysis revealed that fewer people with depression/anxiety previously used psychedelics (p = 0.03), but this group was more likely to agree that the government should fund studies (p = 0.02). More people with addiction disorders previously used psychedelics (p = 0.01), and this group was less likely to agree that the government should fund studies (p = 0.01). Participants with psychotic disorders disagreed that psychedelics increase connection to nature (p = 0.01).

### Possible indication vs contra-indication

Participants with diagnoses with a possible therapeutic indication were more likely to agree that psilocybin may be useful for some mental health disorders (p = 0.003), for depression (p = 0.003), anxiety (p = 0.048), for chronic pain (p = 0.03), psilocybin safe recreationally (p = 0.03), psilocybin increases connection to nature (p < 0.001), psilocybin should be tested for medicinal value (p = 0.009), granted medical treatment status (p = 0.004), would accept psilocybin if doctor recommended (p = 0.016), and willing to come off medications (p = 0.08). Those with diagnoses with a possible therapeutic indication were more likely to disagree that psychedelics are unsafe even under medical supervision (p = 0.009) (Fig. 4B; Table S10).

### Influence of education

There were no statistical differences in attitudes according to education level (Table S11).

### Influence of employment

When students and retired participants were excluded, those who were unemployed disagreed that psilocybin could be used in the treatment of drug and alcohol disorders when compared with those that were in employment (p = 0.01). There were no other differences between the two groups (Table S12).

### Concerns about discontinuing medication to accept psilocybin therapy

**Fig. 5:**
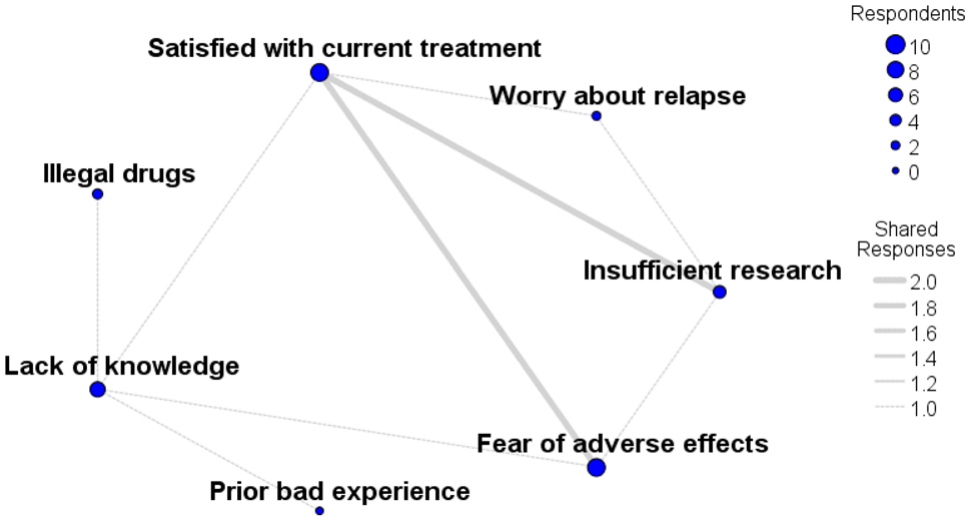
Concerns about discontinuing medication to accept psilocybin therapy. Web diagram of those who answered the question why they would not come off their medication to accept psilocybin therapy (n = 29). The most frequent answer cited was a fear of adverse effects (n = 10). Other reasons cited were satisfaction with their current treatment regime (n = 9), lack of knowledge (n = 7), insufficient available research (n = 5), illegal status of psychedelics (n = 3), worries about relapse of mental illness (n = 2), prior history of addiction (n = 1), and prior negative experience (n = 1). Blue dot size represents the number of participant responses in that category. The grey lines connecting two blue dots represent shared responses, and the line width reflects the number of participants who shared response categories

Of those who answered the question of why they would not come off their medication to accept psilocybin therapy (n = 29), the most frequent answer cited was a fear of adverse effects (n = 10). Other reasons cited were satisfaction with their current treatment regime (n = 9), lack of knowledge (n = 7), insufficient research available (n = 5), current illegal status of psychedelics (n = 3), worries about relapse of mental illness (n = 2), prior history of addiction (n = 1), and prior negative experience (n = 1) (Fig. 5).

## Discussion

This survey provided an insight into the attitudes of mental health service users to psychedelics and psilocybin therapy. The majority (72%) of participants approved of further research into psilocybin therapy, whereas 59% thought it should be approved as a medical treatment, and 52% agreed the government should fund further psilocybin studies. Approximately, one-third (35%) agreed that psilocybin could be useful for some mental disorders, 30% thought it might be useful for their own mental health problem, and just over half (54%) would accept psilocybin therapy if a doctor recommended it and would be willing to come off medications (55%). Younger people, those who had previously used psychedelics, and those with no religious beliefs held more favourable attitudes to psilocybin therapy.

Our survey identified a degree of therapeutic misalignment in that a minority of the total sample (15%) agreed that psilocybin would be useful for psychotic disorders and BPAD, including 5% of the total sample who had those conditions (2 people with BPAD and 3 people with psychosis), whereas 18% were neutral on the topic. Considering psychedelics can exacerbate psychosis and mania [38, 62, 63], this highlights a public health opportunity for enhanced shared scientific understanding of the potential risks of psychedelics.

The rate of lifetime psychedelic use in our survey was high at 35%, which mostly comprised of psilocybin (27%). In keeping with the well-established findings that men are more likely than women to use almost all types of illicit drugs [64], the rates of psychedelic use and most other substances were higher in males. The total sample rate of psilocybin use in the last year (7%) was much higher than the 0.5% rate from previous nationally representative surveys in Ireland [54] and more recently in the UK [53]. In contrast, and unsurprisingly given the sampling bias, our rate was considerably lower than the 55% reported in the most recent GDS 2020 survey [49].

Similarly, our 2% microdosing rate was much lower than the Irish data from the GDS which reported a remarkable 27.5% psilocybin microdosing rate and a 22% LSD microdosing rate over the last 12 months [49]. Despite the postulated mood and cognitive benefits, and increasing popularity [65] of psychedelic microdosing [66–70], clear benefits have not materialized from controlled trials [71–73]. Furthermore, a recently published and commendable citizen scientist effort to investigate microdosing using a self-blinding approach also failed to show clear benefits over placebo [74]. While it is feasible that microdosing may play some role in the precise-personalized-psychedelic therapy paradigm [2, 75], this is not supported by the currently available clinical data.

Conversely, there is accumulating clinical data indicating a transdiagnostic antidepressant [3, 5, 6], anxiolytic [11, 76], and anti-addictive [8–10] therapeutic action of higher doses of psilocybin administered in the context of psychological support. Results from ongoing phase 2 RCTs [77, 78] will determine whether psilocybin therapy will progress to phase 3 trials and likely follow esketamine/ketamine and MDMA therapy for PTSD [79] into the psychiatric clinic.

Curiously, the highest level of agreement for therapeutic indication in our survey was for chronic pain (34%), which has the least amount of clinical data [80]. This was followed by 30% for depression, 24% for anxiety, and 20% for addiction disorders. Overall, approximately one-third (35%) of our participants agreed that psilocybin could be useful for some mental disorders, similar to the attitudes of American college students [55] and lower than those of American psychiatrists at 42.5% [57]. Interestingly, and in keeping with males and younger people having more favourable attitudes to psilocybin therapy, male psychiatrists and early career stage trainees also reported more favourable attitudes towards the potential therapeutic use of psychedelics [57].

Given the possibility that psychiatry may encounter the prospect of psychedelic therapy in the near future [4, 14], it would be interesting to survey Irish psychiatrists’ and psychotherapists attitudes to the evolving field of translational psychedelic science, especially considering the previous contentious relationship between psychiatry and psychedelic therapy.

An intriguing finding from our study indicates that religiosity tempered attitudes to psilocybin therapy. Influenced by pre-existing beliefs, the psychedelic experience is often described in religious terms [81, 82]. Indeed, the Aztec name for some species of psilocybin mushroom was Teonanacatl, translated as “flesh of the gods” or “God’s flesh” [83, 84]. In our sample, participants without religious beliefs had more favourable attitudes to psilocybin therapy than those with religious beliefs and were more likely to agree that psilocybin could increase people’s connection to nature and to other people. We eagerly await results from an open label study investigating the impact of psilocybin-facilitated experiences on psychological functioning, spirituality, health, well-being, and prosocial attitudes in professional religious leaders (NCT02421263). Regardless of one’s position on the multidimensional secular-spiritual belief spectrum, the evidence base and therapeutic application of psychedelic therapy should be grounded in an empirically based scientific framework [41, 61].

One fifth of our sample deemed psychedelics unsafe even under medical supervision, just below the 25% of American Psychiatrists [57]. A similar proportion (20%) viewed psilocybin as addictive, which is not in keeping with the currently available pre-clinical and clinical data [16, 85–88]. In addition to concerns about addiction, our qualitative analysis suggests that participants were concerned with adverse effects, lack of information, insufficient research, illegality, and relapse if medications discontinued. In terms of lack of information, which was reflected more broadly in many people answering “don’t know” or “neutral” to questions; it is important to acknowledge that this survey was conducted prior to results from large scale RCTs. Given the lack of RCT data, this may be appropriate, and further surveys will be able to chart the knowledge/attitude trajectory when more robust data is available.

The concerns highlighted in our survey overlap with concerns from a previous GDS survey (2019) of 85,000 people which highlighted concerns related to “brain damage” and “bad trips” [58]. The same GDS 2019 reported that 59% of people who previously used psychedelics said they would accept it for depression or PTSD compared to only 18% of those surveyed who have never used psychedelics [58]. Similarly, participants in our study who had previously used psychedelics had more favourable attitudes to psilocybin therapy for depression and anxiety disorders, but also psychotic disorders. It is worth noting that while 55% of our total sample would accept psilocybin therapy if their doctor recommended it, 25% were neutral. It is reasonable to speculate that a higher proportion of people would accept psilocybin therapy if their doctor actually recommended it, rather than a hypothetical question in survey.

Similar to other surveys, level of support for further medical exploration and research of the therapeutic potential of psilocybin in our study was high at 72%. This was marginally lower than the 80.5% of American psychiatrists [57] and the 84% of American college students who supported the concept [56]. Somewhat surprisingly given the 72% agreement that psilocybin should be tested for medical purposes, only 52% of our total sample agreed that the government should fund psilocybin studies, though 29% were neutral. Those participants with depression/anxiety disorders, while having lower levels of previous psychedelic use, were more likely to agree that the government should fund studies of psilocybin therapy. Unfortunately, the survey did not capture attitudes as to who, other than the government, should fund such endeavours. Regardless, the best interests of the public should be central to the trajectory of psychedelic therapy.

Almost 60% of our participants agreed that psilocybin should be approved as a medical treatment. It is interesting to note that psilocybin and LSD were prescribed by psychiatrists throughout the USA and Europe, until classified as Schedule I in the United Nations Convention on Drugs in 1967 [89]. Psilocybin was granted breakthrough therapy designation for Treatment-Resistant Depression in October 2018 by the FDA. Recently, voters in Oregon in the USA decriminalized psilocybin and granted it treatment status for therapeutic use in licensed medical facilities, under the supervision of trained professionals. Indeed, legal reclassification would expedite the clinical research programme needed to integrate high-quality psychedelic therapy into public psychiatry for the benefit of those with non-psychotic mental health disorders [90, 91].

A smaller proportion (38%) of our sample were in favour of legalizing recreational psilocybin, with younger participants and those with previous psychedelic use having more liberal views on the legal status of psilocybin. In addition to more liberal views on legalization, younger people were more likely to have used psychedelics and to report themselves as knowledgeable about psychedelics. This is all the more relevant in the context of increasing recreational drug use, including self-medication, and the limited impact of prohibition on reducing supply [92, 93]. Our survey did not enquire as to the source of substance acquisition, though the 16.3% of Irish respondents that used the dark web over the last 12 months reported in the 2017 GDS [94] is likely to have increased.

Data from the GDS 2020 highlights the potential detrimental impact of unsupervised or poorly supervised psychedelic experiences on individuals, particularly those with underlying vulnerabilities. Prior to psychedelic use, 40% of people did not undergo any preparatory or integration sessions [49], suggesting a careless attitude, with an associated increased level of risk of adverse events. Furthermore, 4.2% of those using any recreational psychedelics (including ayahuasca and DMT), for emotional distress or psychiatric conditions, sought emergency medical treatment over the last 12 months, including people with self-reported psychotic (0.2%) and bipolar (2.1%) disorders [49]. The rate of emergency medical treatment was 1% for LSD and 0.2% for psilocybin [94].

The precise trajectory of psychedelic science and its translational corollary, psychedelic therapy, is not yet clear. A system-based precise-personalized psychedelic therapy paradigm that incorporates high-quality therapeutic support has the potential to optimize therapeutic outcomes while mitigating risks [40, 41]. More broadly, a multi-stakeholder, cooperative, open science model [59] that ensures the prioritization of therapeutic outcomes over the potential profitability is likely to lead to the greatest benefits to society and those with mental health problems.

## Conclusions

The majority (72%) supported further research into the therapeutic potential of psilocybin. Younger people, those with previous psychedelic experience, and those with non-religious beliefs were more likely to have favourable attitudes towards psilocybin therapy. This included a small minority with mental health problems that could be exacerbated by psilocybin. Clear public health messaging and communication between researchers, psychiatrists, service users, and the public is necessary for the shared scientific understanding and optimal trajectory of psychedelic science and its translational corollary psychedelic therapy.

### Limitations

We acknowledge, due to non-response bias in the community sample, that it may not be fully representative of total mental health service user population. Our study had a high level of Do not know/Neutral responses. This survey relied on self-reported drug history. Data regarding personal history of mania, violence, suicide attempts, family psychiatric history, reasons for psychedelic use, source of psychedelics, or presentations for emergency medical or psychiatric treatment due to psychedelic use was not recorded. The large number of Chi square tests was not corrected for multiple comparisons. Our diagnoses data lacked specificity to differentiate BPAD I from II, nor phase of current episode.

## Supplementary information

Below is the link to the electronic supplementary material.

Supplementary file1 (DOCX 100 KB)

## References

[1] Nutt D, Carhart-Harris R (2020). The current status of psychedelics in psychiatry. JAMA Psychiat.

[2] Yaden DB, Griffiths RR (2020). The subjective effects of psychedelics are necessary for their enduring therapeutic effects. ACS Pharmacology & Translational Science.

[3] Davis AK, Barrett FS, May DG, Cosimano MP, Sepeda ND, Johnson MW, Finan PH, Griffiths RR (2020). Effects of psilocybin-assisted therapy on major depressive disorder: a randomized clinical trial. JAMA Psychiat.

[4] Carhart-Harris R, Giribaldi B, Watts R, Baker-Jones M, Murphy-Beiner A, Murphy R, Martell J, Blemings A, Erritzoe D, Nutt DJ (2021). Trial of psilocybin versus escitalopram for depression. N Engl J Med.

[5] Carhart-Harris RL, Bolstridge M, Rucker J, Day CM, Erritzoe D, Kaelen M, Bloomfield M, Rickard JA, Forbes B, Feilding A, Taylor D, Pilling S, Curran VH, Nutt DJ (2016). Psilocybin with psychological support for treatment-resistant depression: an open-label feasibility study. Lancet Psychiatry.

[6] Carhart-Harris RL, Bolstridge M, Day CMJ, Rucker J, Watts R, Erritzoe DE, Kaelen M, Giribaldi B, Bloomfield M, Pilling S, Rickard JA, Forbes B, Feilding A, Taylor D, Curran HV, Nutt DJ (2018). Psilocybin with psychological support for treatment-resistant depression: six-month follow-up. Psychopharmacology.

[7] Garcia-Romeu A, Davis AK, Erowid F, Erowid E, Griffiths RR, Johnson MW (2019). Cessation and reduction in alcohol consumption and misuse after psychedelic use. J Psychopharmacol.

[8] DiVito AJ, Leger RF (2020). Psychedelics as an emerging novel intervention in the treatment of substance use disorder: a review. Mol Biol Rep.

[9] Krebs TS, Johansen PO (2012). Lysergic acid diethylamide (LSD) for alcoholism: meta-analysis of randomized controlled trials. J Psychopharmacol.

[10] Bogenschutz MP, Forcehimes AA, Pommy JA, Wilcox CE, Barbosa PC, Strassman RJ (2015). Psilocybin-assisted treatment for alcohol dependence: a proof-of-concept study. J Psychopharmacol.

[11] Moreno FA, Wiegand CB, Taitano EK, Delgado PL (2006). Safety, tolerability, and efficacy of psilocybin in 9 patients with obsessive-compulsive disorder. J Clin Psychiatry.

[12] Spriggs MJ, Kettner H, Carhart-Harris RL (2020). Positive effects of psychedelics on depression and wellbeing scores in individuals reporting an eating disorder. Eating and Weight Disorders - Studies on Anorexia, Bulimia and Obesity.

[13] Schindler EAD, Sewell RA, Gottschalk CH, Luddy C, Flynn LT, Lindsey H, Pittman BP, Cozzi NV, D’Souza DC (2020). Exploratory controlled study of the migraine-suppressing effects of psilocybin. Neurotherapeutics.

[14] Inserra A, De Gregorio D, Gobbi G (2021). Psychedelics in psychiatry: neuroplastic, immunomodulatory, and neurotransmitter mechanisms. Pharmacol Rev.

[15] Teixeira PJ, Johnson M, Timmermann C, Watts R, Erritzoe D, Douglass H, Kettner HRCH (2021). Psychedelics and health behavior change. J Psychopharmacol (in press).

[16] Nichols DE (2016). Psychedelics. Pharmacol Rev.

[17] Glennon RA, Titeler M, McKenney JD (1984). Evidence for 5-HT2 involvement in the mechanism of action of hallucinogenic agents. Life Sci.

[18] González-Maeso J, Weisstaub NV, Zhou M, Chan P, Ivic L, Ang R, Lira A, Bradley-Moore M, Ge Y, Zhou Q, Sealfon SC, Gingrich JA (2007). Hallucinogens Recruit Specific Cortical 5-HT2A Receptor-Mediated Signaling Pathways to Affect Behavior. Neuron.

[19] Weber ET, Andrade R (2010). Htr2a Gene and 5-HT(2A) Receptor expression in the cerebral cortex studied using genetically modified mice. Front Neurosci.

[20] Andrade R (2011). Serotonergic regulation of neuronal excitability in the prefrontal cortex. Neuropharmacology.

[21] Burt JB, Preller KH, Demirtaş M, Ji JL, Krystal JH, Vollenweider FX, Anticevic A, Murray JD (2021) Transcriptomics-informed large-scale cortical model captures topography of pharmacological neuroimaging effects of LSD. bioRxiv:2021.2001.2031.429016. 10.1101/2021.01.31.429016 10.7554/eLife.69320 PMC8315798 34313217

[22] Kim K, Che T, Panova O, DiBerto JF, Lyu J, Krumm BE, Wacker D, Robertson MJ, Seven AB, Nichols DE, Shoichet BK, Skiniotis G, Roth BL (2020). Structure of a hallucinogen-activated Gq-Coupled 5-HT2A Serotonin Receptor. Cell.

[23] Stenbæk DS, Madsen MK, Ozenne B, Kristiansen S, Burmester D, Erritzoe D, Knudsen GM, Fisher PM (2020). Brain serotonin 2A receptor binding predicts subjective temporal and mystical effects of psilocybin in healthy humans. J Psychopharmacol.

[24] Dong C, Ly C, Dunlap LE, Vargas MV, Sun J, Hwang IW, Azinfar A, Oh WC, Wetsel WC, Olson DE, Tian L (2021). Psychedelic-inspired drug discovery using an engineered biosensor. Cell.

[25] Singleton SP, Luppi AI, Carhart-Harris RL, Cruzat J, Roseman L, Deco G, Kringelbach ML, Stamatakis EA, Kuceyeski A (2021) LSD flattens the brain’s energy landscape: evidence from receptor-informed network control theory. bioRxiv:2021.2005.2014.444193. 10.1101/2021.05.14.444193

[26] Hesselgrave N, Troppoli TA, Wulff AB, Cole AB, Thompson SM (2021). Harnessing psilocybin: antidepressant-like behavioral and synaptic actions of psilocybin are independent of 5-HT2R activation in mice. Proc Natl Acad Sci.

[27] Morales-Garcia JA, de la Fuente RM, Alonso-Gil S, Rodriguez-Franco MI, Feilding A, Perez-Castillo A, Riba J (2017). The alkaloids of Banisteriopsis caapi, the plant source of the Amazonian hallucinogen Ayahuasca, stimulate adult neurogenesis in vitro. Sci Rep.

[28] Catlow BJ, Song S, Paredes DA, Kirstein CL, Sanchez-Ramos J (2013). Effects of psilocybin on hippocampal neurogenesis and extinction of trace fear conditioning. Exp Brain Res.

[29] Ly C, Greb AC, Cameron LP, Wong JM, Barragan EV, Wilson PC, Burbach KF, Soltanzadeh Zarandi S, Sood A, Paddy MR, Duim WC, Dennis MY, McAllister AK, Ori-McKenney KM, Gray JA, Olson DE (2018). Psychedelics promote structural and functional neural plasticity. Cell Rep.

[30] Cameron LP, Tombari RJ, Lu J, Pell AJ, Hurley ZQ, Ehinger Y, Vargas MV, McCarroll MN, Taylor JC, Myers-Turnbull D, Liu T, Yaghoobi B, Laskowski LJ, Anderson EI, Zhang G, Viswanathan J, Brown BM, Tjia M, Dunlap LE, Rabow ZT, Fiehn O, Wulff H, McCorvy JD, Lein PJ, Kokel D, Ron D, Peters J, Zuo Y, Olson DE (2020). A non-hallucinogenic psychedelic analogue with therapeutic potential. Nature.

[31] Ly C, Greb AC, Vargas MV, Duim WC, Grodzki ACG, Lein PJ, Olson DE (2020). Transient stimulation with psychoplastogens is sufficient to initiate neuronal growth. ACS Pharmacology & Translational Science.

[32] Mason NL, Kuypers KPC, Müller F, Reckweg J, Tse DHY, Toennes SW, Hutten N, Jansen JFA, Stiers P, Feilding A, Ramaekers JG (2020). Me, myself, bye: regional alterations in glutamate and the experience of ego dissolution with psilocybin. Neuropsychopharmacology.

[33] Vollenweider FX, Preller KH (2020). Psychedelic drugs: neurobiology and potential for treatment of psychiatric disorders. Nat Rev Neurosci.

[34] Carhart-Harris RL (2019). How do psychedelics work?. Curr Opin Psychiatry.

[35] Carhart-Harris RL, Roseman L, Bolstridge M, Demetriou L, Pannekoek JN, Wall MB, Tanner M, Kaelen M, McGonigle J, Murphy K, Leech R, Curran HV, Nutt DJ (2017). Psilocybin for treatment-resistant depression: fMRI-measured brain mechanisms. Sci Rep.

[36] Roseman L, Demetriou L, Wall MB, Nutt DJ, Carhart-Harris RL (2018). Increased amygdala responses to emotional faces after psilocybin for treatment-resistant depression. Neuropharmacology.

[37] Kraehenmann R, Preller KH, Scheidegger M, Pokorny T, Bosch OG, Seifritz E, Vollenweider FX (2015). Psilocybin-induced decrease in amygdala reactivity correlates with enhanced positive mood in healthy volunteers. Biol Psychiatry.

[38] Dos Santos RG, Bouso JC, Hallak JEC (2017). Ayahuasca, dimethyltryptamine, and psychosis: a systematic review of human studies. Ther Adv Psychopharmacol.

[39] Leptourgos P, Fortier-Davy M, Carhart-Harris R, Corlett PR, Dupuis D, Halberstadt AL, Kometer M, Kozakova E, LarØi F, Noorani TN, Preller KH, Waters F, Zaytseva Y, Jardri R (2020). Hallucinations Under Psychedelics and in the Schizophrenia Spectrum: An Interdisciplinary and Multiscale Comparison. Schizophr Bull.

[40] Carhart-Harris RL, Wagner AC, Agrawal M, Kettner H, Rosenbaum JF, Gazzaley A, Nutt DJ, Erritzoe D (2021) Can pragmatic research, real-world data and digital technologies aid the development of psychedelic medicine? J Psychopharmacol (Oxford, England):2698811211008567. 10.1177/02698811211008567 10.1177/02698811211008567 PMC8801625 33888025

[41] Kelly JR, Crockett MT, Alexander L, Haran M, Baker A, Burke L, Brennan C, O'Keane V (2021). Psychedelic science in post-COVID-19 psychiatry. Ir J Psychol Med.

[42] Carbonaro TM, Bradstreet MP, Barrett FS, MacLean KA, Jesse R, Johnson MW, Griffiths RR (2016). Survey study of challenging experiences after ingesting psilocybin mushrooms: acute and enduring positive and negative consequences. J Psychopharmacol.

[43] RANZCP (2020) The Royal Australian and New Zealand College of Psychiatrists (RANZCP). Therapeutic use of psychedelic substances. https://www.ranzcp.org/files/resources/college_statements/clinical_memoranda/cm-therapeutic-use-of-psychedelics.aspx

[44] Kelly JR, Baker A, Babiker M, Burke L, Brennan C, O’Keane V (2019) The psychedelic renaissance: the next trip for psychiatry? Ir J Psychol Med 1–5. 10.1017/ipm.2019.39 10.1017/ipm.2019.39 31543078

[45] Nutt D, Erritzoe D, Carhart-Harris R (2020). Psychedelic Psychiatry's Brave New World. Cell.

[46] Roche A, Kostadinov V, Chapman J, McEntee A (2020). Have decreases in young workers’ risky drinking resulted in an increase in illicit drug use?. Health Promot J Austr.

[47] Palamar JJ, Le A (2018). Trends in DMT and other tryptamine use among young adults in the United States. Am J Addict.

[48] Yockey RA, Vidourek RA, King KA (2020). Trends in LSD use among US adults: 2015–2018. Drug Alcohol Depend.

[49] Winstock AR, Timmermann C, Davis E, Maier LJ, Zhuparris A, Ferris JA, Barratt MJ, Kuypers KPC (2020) Global Drugs Survey (GDS) 2020 Psychedelics Key Findings Report

[50] U.S. Department of Health and Human Services SAaMHSAS, Center for Behavioral Health Statistics and Quality (2019) (2019) Key Substance Use and Mental Health Indicators in the United States: results from the 2018 National Survey on Drug Use and Health. https://www.samhsa.gov/data/sites/default/files/cbhsq-reports/NSDUHNationalFindingsReport2018/NSDUHNationalFindingsReport2018.pdf

[51] Killion B, Hai AH, Alsolami A, Vaughn MG, Sehun OhP, Salas-Wright CP (2021). LSD use in the United States: trends, correlates, and a typology of us. Drug Alcohol Depend.

[52] Addiction. EMCfDaD (2020) Impact of COVID-19 on patterns of drug use and drug-related harms in Europe, EMCDDA Trendspotter briefing

[53] Wales DMFftCSfEa (2019) Drugs misuse: findings from the 2018/19 crime survey for England and Wales. https://www.gov.uk/government/statistics/drug-misuse-findings-from-the-2018-to-2019-csew

[54] PHIRB (2011) National Advisory Committee on Drugs (NACD) & Public Health Information and Research Branch.Drug Use in Irelan

[55] Grant JE, Lust K, Chamberlain SR (2019) Hallucinogen use is associated with mental health and addictive problems and impulsivity in university students. Addict Behav Rep 10. 10.1016/j.abrep.2019.100228 10.1016/j.abrep.2019.100228 PMC6887552 31799366

[56] Wildberger JI, John CN, Hallock RM (2017). Perceptions of the medicinal value of hallucinogenic drugs among college students.

[57] Barnett BS, Siu WO, Pope HG (2018). A survey of American psychiatrists’ attitudes toward classic hallucinogens. J Nerv Ment Dis.

[58] Winstock AR, Johnson MW (2019) Global drugs survey: the psychedelic revolution in psychiatry and why patient opinion matters so much. https://www.globaldrugsurvey.com/gds-2019/gds2019-the-psychedelic-revolution-in-psychiatry-and-why-patient-opinion-matters-so-much/

[59] Statement on open science and open praxis with psilocybin, MDMA, and similar substances (2017) https://files.csp.org/open

[60] Yaden DB, Yaden ME, Griffiths RR (2020). Psychedelics in psychiatry—keeping the renaissance from going off the rails. JAMA Psychiat.

[61] Johnson MW (2020). Consciousness, religion, and gurus: pitfalls of psychedelic medicine. ACS Pharmacology & Translational Science.

[62] Szmulewicz AG, Valerio MP, Smith JM (2015). Switch to mania after ayahuasca consumption in a man with bipolar disorder: a case report. Int J Bipolar Disord.

[63] Brown T, Shao W, Ayub S, Chong D, Cornelius C (2017). A Physician’s attempt to self-medicate bipolar depression with N, N-dimethyltryptamine (DMT). J Psychoactive Drugs.

[64] Abuse NIoD (2020) Substance Use in Women Research Report

[65] Rosenbaum D, Weissman C, Anderson T, Petranker R, Dinh-Williams LA, Hui K, Hapke E (2020). Microdosing psychedelics: demographics, practices, and psychiatric comorbidities. J Psychopharmacol.

[66] Fadiman J, Korb S (2019). Might microdosing psychedelics be safe and beneficial?. An initial exploration J Psychoactive Drugs.

[67] Kuypers KP, Ng L, Erritzoe D, Knudsen GM, Nichols CD, Nichols DE, Pani L, Soula A, Nutt D (2019). Microdosing psychedelics: More questions than answers? An overview and suggestions for future research. J Psychopharmacol.

[68] Polito V, Stevenson RJ (2019). A systematic study of microdosing psychedelics. PLoS ONE.

[69] Anderson T, Petranker R, Rosenbaum D, Weissman CR, Dinh-Williams LA, Hui K, Hapke E, Farb NAS (2019). Microdosing psychedelics: personality, mental health, and creativity differences in microdosers. Psychopharmacology.

[70] Anderson T, Petranker R, Christopher A, Rosenbaum D, Weissman C, Dinh-Williams LA, Hui K, Hapke E (2019). Psychedelic microdosing benefits and challenges: an empirical codebook. Harm Reduct J.

[71] Bershad AK, Schepers ST, Bremmer MP, Lee R, de Wit H (2019). Acute subjective and behavioral effects of microdoses of lysergic acid diethylamide in healthy human volunteers. Biol Psychiat.

[72] Yanakieva S, Polychroni N, Family N, Williams LTJ, Luke DP, Terhune DB (2019). The effects of microdose LSD on time perception: a randomised, double-blind, placebo-controlled trial. Psychopharmacology.

[73] Family N, Maillet EL, Williams LTJ, Krediet E, Carhart-Harris RL, Williams TM, Nichols CD, Goble DJ, Raz S (2020). Safety, tolerability, pharmacokinetics, and pharmacodynamics of low dose lysergic acid diethylamide (LSD) in healthy older volunteers. Psychopharmacology.

[74] Szigeti B, Kartner L, Blemings A, Rosas F, Feilding A, Nutt DJ, Carhart-Harris RL, Erritzoe D (2021) Self-blinding citizen science to explore psychedelic microdosing. eLife 10:e62878. 10.7554/eLife.62878 10.7554/eLife.62878 PMC7925122 33648632

[75] Olson DE (2020). The Subjective Effects of Psychedelics May Not Be Necessary for Their Enduring Therapeutic Effects. ACS Pharmacology & Translational Science.

[76] Weston NM, Gibbs D, Bird CIV, Daniel A, Jelen LA, Knight G, Goldsmith D, Young AH, Rucker JJ (2020). Historic psychedelic drug trials and the treatment of anxiety disorders. Depress Anxiety.

[77] Medicine USNLo (2021) The safety and efficacy of psilocybin in participants with treatment resistant depression (P-TRD) (ClinicalTrials.gov). https://clinicaltrials.gov/ct2/show/NCT03775200?term=PSILOCYBIN

[78] Medicine USNLo (2021) A Study of Psilocybin for Major Depressive Disorder (MDD). https://clinicaltrials.gov/ct2/show/NCT03866174?term=PSILOCYBIN

[79] Mitchell JM, Bogenschutz M, Lilienstein A, Harrison C, Kleiman S, Parker-Guilbert K, Ot’alora G M, Garas W, Paleos C, Gorman I, Nicholas C, Mithoefer M, Carlin S, Poulter B, Mithoefer A, Quevedo S, Wells G, Klaire SS, van der Kolk B, Tzarfaty K, Amiaz R, Worthy R, Shannon S, Woolley JD, Marta C, Gelfand Y, Hapke E, Amar S, Wallach Y, Brown R, Hamilton S, Wang JB, Coker A, Matthews R, de Boer A, Yazar-Klosinski B, Emerson A, Doblin R,  (2021). MDMA-assisted therapy for severe PTSD: a randomized, double-blind, placebo-controlled phase 3 study. Nat Med.

[80] Castellanos JP, Woolley C, Bruno KA, Zeidan F, Halberstadt A, Furnish T (2020) Chronic pain and psychedelics: a review and proposed mechanism of action. Regional Anesthesia & Pain Medicine:rapm-2020–101273. 10.1136/rapm-2020-101273 10.1136/rapm-2020-101273 32371500

[81] Watts A (1968). Psychedelics and Religious Experience. Calif Law Rev.

[82] Griffiths RR, Hurwitz ES, Davis AK, Johnson MW, Jesse R (2019). Survey of subjective “God encounter experiences”: comparisons among naturally occurring experiences and those occasioned by the classic psychedelics psilocybin, LSD, ayahuasca, or DMT. PLoS ONE.

[83] Schultes RE, Hofmann A, Rätsch C (2001). Plants of the gods: their sacred, healing, and hallucinogenic powers.

[84] Guzmán G (1983) The genus Psilocybe : a systematic revision of the known species including the history, distribution, and chemistry of the hallucinogenic species. J. Cramer, Vaduz [Liechtenstein]

[85] Nutt D, King LA, Saulsbury W, Blakemore C (2007). Development of a rational scale to assess the harm of drugs of potential misuse. Lancet.

[86] Bonomo Y, Norman A, Biondo S, Bruno R, Daglish M, Dawe S, Egerton-Warburton D, Karro J, Kim C, Lenton S, Lubman DI, Pastor A, Rundle J, Ryan J, Gordon P, Sharry P, Nutt D, Castle D (2019). The Australian drug harms ranking study. J Psychopharmacol.

[87] Johnson MW, Griffiths RR, Hendricks PS, Henningfield JE (2018). The abuse potential of medical psilocybin according to the 8 factors of the Controlled Substances Act. Neuropharmacology.

[88] Krebs TS, Johansen PØ (2013). Psychedelics and mental health: a population study. PLoS ONE.

[89] Rucker JJH, Iliff J, Nutt DJ (2018). Psychiatry & the psychedelic drugs. Past, present & future. Neuropharmacology.

[90] Rucker JJ (2015). Psychedelic drugs should be legally reclassified so that researchers can investigate their therapeutic potential. BMJ.

[91] Nutt DJ, King LA, Nichols DE (2013). Effects of Schedule I drug laws on neuroscience research and treatment innovation. Nat Rev Neurosci.

[92] Policy GCOD (2021) https://www.globalcommissionondrugs.org/reports

[93] Werb D, Rowell G, Guyatt G, Kerr T, Montaner J, Wood E (2011). Effect of drug law enforcement on drug market violence: a systematic review. Int J Drug Policy.

[94] Winstock A, Barratt M, Ferris JLM (2017) Global Drug Survey 10.1177/1178221817716391 PMC5595253 28924351

