# Supplementary material for: Psychedelic perceptions: mental health service user attitudes to psilocybin therapy
Source: Ir J Med Sci. 2021 Jun 15;191(3):1385–97. doi: 10.1007/s11845-021-02668-2 (PMC8205319; doi:10.1007/s11845-021-02668-2)
Supplement: Supplementary file 1 — Supplementary file1 (DOCX 100 KB) [file 11845_2021_2668_MOESM1_ESM.docx]

**Psychedelic Perceptions: Mental Health Service User Attitudes to Psilocybin Therapy**

**Supplemental Information**

| **Table S1** | **Total sample Attitudes** |
| --- | --- |
| **Table S2** | **Total sample previous drug use** |
| **Table S3** | **Attitudes by previous psychedelic use** |
| **Table S4** | **Attitudes by previous psychedelic use** (net agree) |
| **Table S5** | **Previous substance use and Attitudes by Gender** |
| **Table S6** | **Attitudes by Age** |
| **Table S7** | **Attitudes by Religion** |
| **Table S8** | **Attitudes by Diagnosis** |
| **Table S9** | **Attitudes by Diagnosis** (net agree) |
| **Table S10** | **Attitudes by possible indication vs contra-indicated diagnoses** |
| **Table S11** | **Attitudes by Education** |
| **Table S12** | **Attitudes by Employment** (excluding retired/student) |
|  | **Questionnaire** |

**Table S1: Total sample Attitudes**

|  | **strongly disagree** (%) | **disagree** (%) | **neutral** (%) | **agree** (%) | **strongly agree** (%) |
| --- | --- | --- | --- | --- | --- |
| **Psilocybin with psychological support shows promise in some mental disorders** | 6.3 | 9.5 | 48.4 | 21.1 | 14.7 |
| Psilocybin with psychological support can be a therapeutic tool for those with | | | | | |
| Depression | 6.3 | 6.3 | 56.8 | 13.7 | 16.8 |
| Anxiety | 8.4 | 9.5 | 57.9 | 13.7 | 10.5 |
| To aid in smoking cessation | 7.3 | 10.4 | 70.8 | 7.3 | 4.2 |
| Chronic pain | 0 | 6.2 | 59.8 | 25.8 | 8.2 |
| Eating disorders | 2.1 | 9.3 | 75.3 | 9.3 | 4.1 |
| Drug or alcohol addiction | 3.3 | 15.4 | 61.5 | 13.2 | 6.6 |
| Psychotic disorders such as schizophrenia | 6.3 | 10.4 | 68.8 | 9.4 | 5.2 |
| **Attitudes to the safety & legal aspect of psilocybin** | | | | | |
| Psilocybin should be tested for medicinal value | 2.1 | 5.2 | 20.8 | 29.2 | 42.7 |
| Psilocybin should be granted medical treatment status | 0 | 7.4 | 34.0 | 29.8 | 28.7 |
| The government should fund studies to explore medicinal uses for Psilocybin | 5.2 | 14.4 | 28.9 | 23.7 | 27.8 |
| Psilocybin can be safely enjoyed when used recreationally | 11.6 | 13.7 | 38.9 | 27.4 | 8.4 |
| Psychedelics should be illegal for recreational purposes | 11.3 | 26.8 | 32.0 | 23.7 | 6.2 |
| Psychedelics are unsafe even under medical supervision | 12.4 | 28.9 | 39.2 | 13.4 | 6.2 |
| **Attitudes and Acceptability** | | | | | |
| Psilocybin with psychological support would be useful for my mental health problem | 6.2 | 12.4 | 51.5 | 14.4 | 15.5 |
| I would accept Psilocybin with psychological support if a doctor recommended it | 7.2 | 13.4 | 24.7 | 32.0 | 22.7 |
| I would be willing to come off my medications to accept Psilocybin with psychological support | 11.2 | 16.3 | 13.3 | 30.6 | 19.4^a^ |
| I would say I am knowledgeable about psychedelic drugs | 20.2 | 24.5 | 20.2 | 28.7 | 6.4 |
| I would say I am knowledgeable about magic mushrooms | 23.4 | 31.9 | 19.1 | 20.2 | 5.3 |
| Psilocybin can increase people’s connection to nature | 2.1 | 8.2 | 49.5 | 20.6 | 19.6 |
| Psilocybin can increase people’s connection to other people | 1.0 | 14.4 | 50.5 | 21.6 | 12.4 |
| Psilocybin can lead to a mystical experience | 1.0 | 2.1 | 45.4 | 38.1 | 13.4 |
| Psilocybin can be addictive | 3.2 | 17.9 | 46.3 | 25.3 | 7.4 |
| a Total does not add up to 100% as 9.2% answered not applicable | | | | | |

**Table S2: Total sample previous drug use**

| **Drug** | **Lifetime** (%) | **Last 12 months** (%) | **Last month** (%) | **Age first use**  (mean, SD) | **Age last use**  (mean, SD) |
| --- | --- | --- | --- | --- | --- |
| Psilocybin | 27.3 | 7.1 | 1.0 | 21.24 (8.11) | 26.59 (8.67) |
| LSD | 21.4 | 3.1 | 1.0 | 19.42 (3.40) | 21.94 (4.28) |
| DMT | 6.1 | 4.1 | 0 | 24.40 (8.17) | 26.20 (7.08) |
| Mescaline | 1.0 | 1.0 | 0 | 23.00 | 23.00 |
| Salvia | 8.2 | 6.1 | 0 | 18.20 (1.30) | 24.25 (5.56) |
| Microdosing | 2.0 | 2.0 | 0 | 26.00 | 26.00 |
| Ketamine | 21.6 | 8.3 | 0 | 23.16 (6.17) | 24.12 (6.25) |
| Tobacco | 72.2 | 40.9 | 30.7 | 16.15 (6.18) | 33.04 (16.10) |
| Alcohol | 88.8 | 58.0 | 37.1 | 15.70 (2.03) | 36.00 (12.62) |
| Cannabis | 57.1 | 22.9 | 12.5 | 18.00 (4.66) | 28.83 (9.46) |
| Cocaine | 42.4 | 19.4 | 7.1 | 22.56 (6.92) | 29.18 (8.25) |
| Crack cocaine | 4.0 | 2.0 | 0 | 23.67 (9.86) | 24.50 (7.85) |
| Ecstasy | 36.4 | 7.1 | 2.0 | 19.97 (4.51) | 23.90 (6.36) |
| MDMA | 26.3 | 7.1 | 1.0 | 21.80 (5.91) | 25.53 (5.86) |
| Amphetamines | 16.2 | 0 | 0 | 18.91 (3.36) | 21.73 (5.15) |
| Methamphetamines | 5.1 | 1.0 | 0 | 17.00 (1.00) | 21.25 (4.99) |
| Barbiturates | 1.0 | 1.0 | 0 | 35.00 | 35.00 |
| Benzodiazepines | 12.1 | 5.1 | 4.1 | 27.33 (9.11) | 30.09 (10.32) |
| GBL/GHB | 0 | 0 | 0 | NA | NA |
| Hypnotics | 4.0 | 1.0 | 1.0 | 33.50 (14.84) | 36.50 (19.09) |
| Volatile inhalants | 5.1 | 2.0 | 1.0 | 17.75 (4.27) | 20.00 (3.16) |
| Opioids | 5.1 | 2.0 | 1.0 | 19.00 (3.31) | 38.33 (2.51) |
| Anabolic Steroids | 1.0 | 0 | 0 | NA | NA |
| Other | 1.0 | 1.0 | 0 | NA | NA |

**Table S3: Attitudes by previous psychedelic use**

|  | | **strongly disagree (%)** | **disagree (%)** | **neutral (%)** | | **agree (%)** | **strongly agree (%)** | **NA(%)** |
| --- | --- | --- | --- | --- | --- | --- | --- | --- |
| **Psilocybin with psychological support would be useful for my mental health problem** | |  |  |  |  | |  |  |
|  | Prior use | 0 | 11.4 | 37.1 | 17.1 | | 34.3 |  |
|  | No prior use | 8.2 | 13.1 | 60.7 | 13.1 | | 4.9 |  |
| **I would accept Psilocybin with psychological support if a doctor recommended it** | |  |  |  |  | |  |  |
|  | Prior use | 2.9 | 8.6 | 5.7 | 40 | | 42.9 |  |
|  | No prior use | 8.2 | 16.4 | 36.1 | 27.9 | | 11.5 |  |
| **I would be willing to come off my medications to accept Psilocybin with psychological support** | |  |  |  |  | |  |  |
|  | Prior use | 2.9 | 11.4 | 2.9 | 37.1 | | 34.3 | 11.4 |
|  | No prior use | 16.1 | 17.7 | 19.4 | 27.4 | | 11.3 | 8.1 |
| **I would say I am knowledgeable about psychedelic drugs** | |  |  |  |  | |  |  |
|  | Prior use | 2.9 | 26.5 | 20.6 | 41.2 | | 8.8 |  |
|  | No prior use | 2.9 | 26.5 | 20.6 | 41.2 | | 8.8 |  |
| **I would say I am knowledgeable about magic mushrooms** | |  |  |  |  | |  |  |
|  | Prior use | 0 | 36.4 | 21.2 | 30.3 | | 12.1 |  |
|  | No prior use | 36.7 | 30 | 18.3 | 13.3 | | 1.7 |  |
| **Psilocybin can increase people’s connection to nature** | |  |  |  |  | |  |  |
|  | Prior use | 0 | 2.9 | 28.6 | 28.6 | | 40 |  |
|  | No prior use | 3.3 | 9.8 | 62.3 | 16.4 | | 8.2 |  |
| **Psilocybin can increase people’s connection to other people** | |  |  |  |  | |  |  |
|  | Prior use | 2.9 | 14.3 | 28.6 | 28.6 | | 25.7 |  |
|  | No prior use | 0 | 11.1 | 63.9 | 18 | | 4.9 |  |
| **Psilocybin can lead to a mystical experience** | |  |  |  |  | |  |  |
|  | Prior use | 0 | 2.9 | 14.3 | 51.4 | | 31.4 |  |
|  | No prior use | 1.6 | 1.6 | 62.3 | 31.1 | | 3.3 |  |
| **Psilocybin can be addictive** | |  |  |  |  | |  |  |
|  | Prior use | 14.7 | 47.1 | 20.6 | 14.7 | | 2.9 |  |
|  | No prior use | 3.3 | 13.3 | 61.7 | 20 | | 1.7 |  |

**Table S4: Attitudes by previous psychedelic use** (net agree)

|  | **Never used psychedelics** count (within group net agree %, excluding neutral) | | **Previous Psychedelics**  count (within group net agree %, excluding neutral) | | Pearson Chi-squared p value |
| --- | --- | --- | --- | --- | --- |
| Knowledgeable about psychedelics | 32 (66.6) | 16 (33.3) | 10 (37) | 17 (63) | *.013** |
| Knowledgeable about magic mushrooms | 40 (80) | 10 (20) | 12 (46.2) | 14 (53.8) | *.003** |
| Psilocybin for some mental health disorders | 11 (42.3) | 15 (57.7) | 4 (17.4) | 19 (82.6) | 0.059 |
| Psilocybin for depression | 9 (42.9) | 12 (57.1) | 3 (15) | 17 (85) | *0.050** |
| Psilocybin for anxiety | 13 (61.9) | 8 (38.1) | 4 (21.1) | 15 (78.9) | *.009*** |
| Psilocybin for smoking cessation | 11 (73.6) | 4 (26.7) | 6 (46.2) | 7 (53.8) | 0.142 |
| Psilocybin for psychotic disorders | 11 (78.6) | 3 (21.4) | 5 (31.3) | 11 (68.7) | .010* |
| Psilocybin for drug and alcohol addiction | 11 (57.9) | 8 (42.1) | 6 (37.5) | 10 (62.5) | .229 |
| Psilocybin for chronic pain | 4 (20) | 16 (80) | 2 (10.5) | 17 (89.5) | .41 |
| Psilocybin for eating disorders | 5 (50) | 5 (50) | 6 (42.9) | 8 (57.1) | .729 |
| Psilocybin increases connection to nature | 9 (37.5) | 15 (62.5) | 1 (4) | 24 (96) | *.004*** |
| Psilocybin increases connection to others | 9 (39.1) | 14 (60.9) | 6 (24) | 19 (76) | .259 |
| Psilocybin can lead to mystical experiences | 2 (8.7) | 21 (91.3) | 1 (3.3) | 29 (96.7) | .402 |
| Psychedelics unsafe even under medical supervision | 18 (58.1) | 13 (41.9) | 22 (78.6) | 6 (21.4) | .092 |
| Psilocybin should be tested for medicinal value | 6 (13.6) | 38 (86.4) | 1 (3.1) | 31 (96.9) | .11 |
| Psilocybin should be illegal recreational use | 15 (38.5) | 24 (61.5) | 22 (81.5) | 5 (18.5) | *.001*** |
| Psilocybin can be safely used recreationally | 14 (48.3) | 15 (51.7) | 10 (34.5) | 19 (65.5) | .286 |
| Psilocybin can be addictive | 10 (41.7) | 14 (58.3) | 21 (77.8) | 6 (22.2) | *.008*** |
| Government should fund psilocybin studies | 8 (20.5) | 31 (79.5) | 11 (36.7) | 19 (63.3) | .136 |
| Psilocybin granted medical treatment status | 6 (17.6) | 28 (82.4) | 1 (3.6) | 27 (96.4) | .081 |
| Psilocybin would benefit my condition | 14 (56) | 11 (44) | 4 (18.2) | 18 (81.8) | *.008*** |
| Would accept psilocybin if doctor recommended | 16 (40) | 24 (60) | 4 (12.1) | 29 (87.9) | *.008*** |
| Willing to come off medications | 22 (47.8) | 24 (52.2) | 5 (16.7) | 25 (83.3) | *.006*** |

**Table S5. Previous substance use and Attitudes by Gender**

|  | **Females** count (% yes/agree within group | **Males**  count (% yes/agree within group) | Pearson Chi-squared p value |
| --- | --- | --- | --- |
| Previous Psychedelic use | 12 (34.3) | 23 (65.7) | *.011** |
| Previous Psilocybin use | 7 (25.9) | 20 (74.1) | *.002*** |
| Previous LSD | 7 (33.3) | 14 (66.7) | .053 |
| Previous DMT | 1 (16.7) | 5 (83.3) | .073 |
| Previous Mescaline | 0 (0) | 1 (100) | .295 |
| Previous Saliva | 3 (37.5) | 5 (62.5) | .390 |
| Previous psychedelic Microdosing | 0 (0) | 2 (100) | .137 |
| Previous Ketamine | 8 (38.1) | 13 (61.9) | .163 |
| Previous Cannabis | 24 (42.9) | 32 (57.1) | .062 |
| Previous cocaine (powder) | 16 (38.1) | 21 (61.9) | *.022** |
| Previous crack cocaine | 2 (50) | 2 (50) | .951 |
| Previous ecstasy | 12 (33.3) | 24 (66.7) | *.006*** |
| Previous MDMA | 8 (30.8) | 18 (69.2) | *.014** |
| Previous amphetamines | 5 (31.3) | 11 (68.8) | .076 |
| Previous methamphetamines | 2 (40) | 3 (60) | .59 |
| Previous Barbiturates | 0 (0) | 1 (100) | .30 |
| Previous Benzodiazepines | 6 (50) | 6 (50) | .911 |
| Previous Hypnotics | 3 (75) | 1 (25) | .337 |
| Previous GHB/GLB | 0 (0) | 0 (0) | NA |
| Previous Volatile Inhalants | 3 (60) | 2 (40) | .697 |
| Previous opioids | 1 (20) | 4 (80) | .148 |
| Previous anabolic steroids | 0 (0) | 1 (100) | .300 |
| Previous Other Substance | 2 (66.7) | 1 (33,3) | .582 |
| Previous Tobacco | 33 (47.1) | 37 (52.9) | .162 |
| Previous Alcohol | 44 (50.6) | 43 (49.4) | .414 |
|  |  |  |  |
| Knowledgeable about psychedelics | 12 (36.4) | 21 (63.6) | .074 |
| Knowledgeable about magic mushrooms | 8 (33.3) | 16 (66.7) | *.048** |
| Psilocybin for some mental health disorders | 13 (38.2) | 21 (61.8) | .325 |
| Psilocybin for depression | 11 (37.9) | 18 (62.1) | .475 |
| Psilocybin for anxiety | 8 (34.8) | 15 (65.2) | .251 |
| Psilocybin can be addictive | 12 (60) | 8 (40) | .208 |
| Psilocybin safe recreationally | 18 (52.9) | 16 (47.1) | .397 |
| Psilocybin for smoking cessation | 3 (27.3) | 8 (72.7) | .180 |
| Psilocybin for psychotic disorders | 4 (28.6) | 10 (71.4) | .127 |
| Psilocybin for drug and alcohol addiction | 7 (38.9) | 11 (61.1) | .625 |
| Psilocybin for chronic pain | 14 (42.4) | 19 (57.6) | .731 |
| Psilocybin for eating disorders | 5 (38.5) | 8 (61.5) | .916 |
| Psilocybin increases connection to nature | 17 (43.6) | 22 (56.4) | .838 |
| Psilocybin increases connection to others | 15 (45.5) | 18 (54.5) | .430 |
| Psilocybin can lead to mystical experiences | 20 (40) | 30 (60) | .165 |
| Psychedelics unsafe even under medical supervision | 9 (47.4) | 10 (52.6) | .583 |
| Psilocybin illegal for recreational use | 13 (44.8) | 16 (55.2) | .758 |
| Psilocybin should be tested for medicinal value | 32 (46.4) | 37 (56.3) | .206 |
| Government should fund psilocybin studies | 22 (44) | 28 (56) | .802 |
| Psilocybin granted medical treatment status | 21 (38.2) | 34 (61.8) | .335 |
| Psilocybin would benefit my condition | 11 (37.9) | 18 (62.1) | .658 |
| Would accept psilocybin if doctor recommended | 20 (37.7) | 33 (62.3) | .183 |
| Willing to come off medications | 19 (38.8) | 30 (61.2) | .159 |

**Table S6: Attitudes by Age**

| **Age** | **<35yrs**  (n=28)  count (within group % agree, excluding neutral) | **36-49yrs**  (n=41) | **>50yrs**  (n=30) | Pearson Chi-squared p value |
| --- | --- | --- | --- | --- |
| Previous psychedelic use | 12 (34.3) | 20 (57.1) | 3 (8.6) | *0.002*** |
| Knowledgeable about psychedelics | 12 (36.4) | 15 (45.5) | 6 (18.2) | *.016** |
| Knowledgeable about magic mushrooms | 8 (33.3) | 15 (62.5) | 1 (4.2) | *.001*** |
| Psilocybin for some mental health disorders | 12 (35.3) | 15 (44.1) | 7 (20.6) | .197 |
| Psilocybin for depression | 10 (34.5) | 15 (51.7) | 4 (13.8) | .647 |
| Psilocybin for anxiety | 7 (30.4) | 12 (52.2) | 4 (17.4) | .530 |
| Psilocybin addictive | 9 (45) | 8 (40) | 3 (15) | .449 |
| Psilocybin safe recreationally | 15 (44.1) | 16 (47.1) | 3 (8.8) | *.034** |
| Psilocybin for smoking cessation | 3 (27.3) | 6 (54.5) | 2 (18.2) | .533 |
| Psilocybin for psychotic disorders | 6 (42.9) | 7 (50) | 1 (7.) | .300 |
| Psilocybin for drug and alcohol addiction | 6 (33.3) | 9 (50) | 3 (16.7) | .985 |
| Psilocybin for chronic pain | 13 (39.4) | 12 (36.4) | 8 (24.2) | .093 |
| Psilocybin for eating disorders | 4 (30.8) | 6 (46.2) | 3 (23.1) | .407 |
| Psilocybin increases connection to nature | 17 (43.6) | 17 (43.6) | 5 (12.8) | .145 |
| Psilocybin increases connection to others | 11 (33.3) | 18 (54.5) | 4 (12.1) | .993 |
| Psilocybin can lead to mystical experiences | 17 (34) | 25 (50) | 8 (16) | .728 |
| Psychedelics unsafe even under medical supervision | 4 (21.1) | 10 (52.6) | 5 (26.3) | .236 |
| Psilocybin should be tested for medicinal value | 22 (31.9) | 29 (42.0) | 18 (26.1) | .607 |
| Psilocybin illegal recreational use | 2 (6.9) | 13 (44.8) | 14 (48.3) | *.001*** |
| Government should fund psilocybin studies | 15 (30) | 20 (40) | 15 (30) | .453 |
| Psilocybin granted medical treatment status | 19 (34.5) | 23 (41.8) | 13 (23.6) | .327 |
| Psilocybin would benefit my condition | 8 (27.6) | 16 (55.2) | 5 (17.2) | .289 |
| Would accept psilocybin if doctor recommended | 19 (35.8) | 24 (45.3) | 10 (18.9) | .511 |
| Willing to come off medications | 16 (32.7) | 22 (44.9) | 11 (22.4) | .356 |

**Table S7: Attitudes by Religion**

| **Religion** | **No Religion**  count (within group %) | | **Religion** | | Pearson Chi-squared p value |
| --- | --- | --- | --- | --- | --- |
|  | No/disagree | Yes/ agree | No/disagree | Yes/ agree |  |
| Previous psychedelic use | 17 (60.7) | 11 (39.3) | 46 (65.7) | 24 (34.3) | .641 |
| Knowledgeable about psychedelics | 8 (47.1) | 9 (52.9) | 33 (57.9) | 24 (42.1) | .43 |
| Knowledgeable about magic mushrooms | 10 (58.8) | 7 (41.2) | 41(70.7) | 17 (29.3) | .356 |
| Psilocybin for some mental health disorders | 1 (8.3) | 11 (91.7) | 14 (37.8) | 23 (62.2) | .054 |
| Psilocybin for depression | 2 (15.4) | 11 (84.6) | 10 (35.7) | 18 (64.3) | .183 |
| Psilocybin for anxiety | 4 (44.4) | 5 (55.6) | 12 (40) | 18 (60) | .812 |
| Psilocybin addictive | 8 (50) | 8 (50) | 22 (64.7) | 12 (35.3) | .322 |
| Psilocybin safe recreationally | 3 (17.6) | 14 (82.4) | 20 (50) | 20(50) | *.023** |
| Psilocybin for smoking cessation | 1 (25) | 3 (75) | 15 (65.2) | 8 (34.8) | .131 |
| Psilocybin for psychotic disorders | 4 (66.7) | 2 (33.3) | 11 (47.8) | 12 (52.2) | .411 |
| Psilocybin for drug and alcohol addiction | 2 (40) | 3 (60) | 15 (50) | 15 (50) | .679 |
| Psilocybin for chronic pain | 0 (0) | 5 (100) | 6 (17.6) | 28(82.4) | .307 |
| Psilocybin for eating disorders | 2 (40) | 3 (60) | 9 (47.4) | 10 (52.6) | .769 |
| Psilocybin increases connection to nature | 0(0) | 13 (100) | 10 (27.8) | 26 (72.2) | *.033** |
| Psilocybin increases connection to others | 1 (8.3) | 11 (91.7) | 14 (38.9) | 22 (61.1) | *.048** |
| Psilocybin can lead to mystical experiences | 1 (5.9) | 16 (94.1) | 2 (5.7) | 33 (94.3) | .981 |
| Psychedelics unsafe even under medical supervision (% disagree) | 14 (82.4) | 3 (17.6) | 25 (61) | 16 (32.8) | .114 |
| Psilocybin should be tested for medicinal value | 1 (4.2) | 23 (95.8) | 6 (11.8) | 45 (88.2) | .291 |
| Psilocybin illegal recreationally | 15 (71.4) | 6 (28.6) | 22 (48.9) | 23 (51.1) | .086 |
| Government should fund psilocybin studies | 6 (26.1) | 17 (73.9) | 13 (28.3) | 33 (71.1) | .849 |
| Psilocybin granted medical treatment status | 1 (5) | 19 (95) | 6 (14.3) | 36 (85.7) | .280 |
| Psilocybin would benefit my condition | 1 (10) | 9 (90) | 17 (45.9) | 20 (54.1) | *.038** |
| Would accept psilocybin if doctor recommended | 1 (4.8) | 20 (95.2) | 19 (36.5) | 33 (63.5) | *.006*** |
| Willing to come off medications | 4 (17.4) | 19 (82.6) | 23 (43.4) | 30 (56.6) | *.030** |

**Table S8: Attitudes by Diagnosis**

| **Attitudes to therapeutic potential** | | | | | | | | | | | | | |
| --- | --- | --- | --- | --- | --- | --- | --- | --- | --- | --- | --- | --- | --- |
| Psilocybin with psychological support shows promise in some mental disorders | | | | | | | | | | | | | |
|  | **strongly disagree** (count, % within diagnosis) | **disagree** (count, % within diagnosis) | | | | **neutral** (count, % within diagnosis) | **agree** (count, % within diagnosis) | | | **strongly agree** (count, % within diagnosis) | | | |
| Anxiety/Depression | 1 (2.8) | 1 (2.8) | | | | 22 (61.1) | 11 (30.6) | | | 1 (2.8) | | | |
| BPAD | 1 (8.3) | 4 (33.3) | | | | 3 (25.0) | 2 (16.7) | | | 2 (16.7) | | | |
| Psychotic | 3 (21.4) | 2 (14.3) | | | | 5 (35.7) | 2 (14.3) | | | 2 (14.3) | | | |
| PD | 1 (7.1) | 1 (7.1) | | | | 7 (50.0) | 2 (14.3) | | | 3 (21.4) | | | |
| Addiction | 0 (0.0) | 1 (5.6) | | | | 8 (44.4) | 3 (16.7) | | | 6 (33.3) | | | |
| Psilocybin with psychological support can be a therapeutic tool for those with depression | | | | | | | | | | | | | |
| Anxiety/Depression | 0 (0.0) | 0 (0.0) | | | | 26 (72.2) | 9 (25.0) | | | 1 (2.9) | | | |
| BPAD | 1 (8.3) | 3 (25.0) | | | | 4 (33.3) | 1 (8.3) | | | 3 (25.0) | | | |
| Psychotic | 3 (20.0) | 1 (6.7) | | | | 9 (60.0) | 0 (0.0) | | | 2 (13.3) | | | |
| PD | 1 (7.1) | 1 (7.1) | | | | 7 (50.0) | 2 (14.3) | | | 3 (21.4) | | | |
| Addiction | 1 (5.9) | 1 (5.9) | | | | 7 (41.2) | 1 (5.9) | | | 7 (41.2) | | | |
| Psilocybin with psychological support can be a therapeutic tool for those with anxiety | | | | | | | | | | | | | |
| Anxiety/Depression | 1 (2.7) | 2 (5.4) | | | | 27 (73) | 7 (18.9) | | | 0 (0.0) | | | |
| BPAD | 1 (9.1) | 3 (27.3) | | | | 4 (36.4) | 1 (9.1) | | | 2 (18.2) | | | |
| Psychotic | 4 (25.0) | 1 (6.3) | | | | 9 (56.3) | 0 (0.0) | | | 2 (12.5) | | | |
| PD | 1 (7.7) | 1 (7.7) | | | | 8 (61.5) | 1 (7.7) | | | 2 (15.4) | | | |
| Addiction | 1 (5.9) | 2 (11.8) | | | | 7 (41.2) | 3 (17.6) | | | 4 (23.5) | | | |
| Psilocybin with psychological support can be a therapeutic tool to aid in smoking cessation | | | | | | | | | | | | | |
| Anxiety/Depression | 0 (0.0) | 3 (8.1) | | | | 32 (86.5) | 2 (5.4) | | | 0 (0.0) | | | |
| BPAD | 1 (9.1) | 2 (18.2) | | | | 6 (54.5) | 1 (9.1) | | | 1 (9.1) | | | |
| Psychotic | 4 (25) | 1 (6.3) | | | | 10 (62.5) | 0 (0.0) | | | 1 (6.3) | | | |
| PD | 1 (7.1) | 1 (7.1) | | | | 11 (78.6) | 1 (7.1) | | | 0 (0.0) | | | |
| Addiction | 1 (5.9) | 3 (17.6) | | | | 8 (47.1) | 3 (17.6) | | | 2 (11.8) | | | |
| Psilocybin with psychological support can be a therapeutic tool for those with chronic pain | | | | | | | | | | | | | |
| Anxiety/Depression | 0 (0.0) | 1 (2.7) | | | | 27 (73.0) | 24.3 (9) | | | | 0 (0.0) | | |
| BPAD | 0 (0.0) | 1 (8.3) | | | | 8 (66.7) | 2 (16.7) | | | | 1 (8.3) | | |
| Psychotic | 0 (0.0) | 4 (25.0) | | | | 37.5 (6) | 18.8 (3) | | | | 18.8 (3) | | |
| PD | 0 (0.0) | 0.0 (0) | | | | 57.1 (8) | 35.7 (5) | | | | 7.1 (1) | | |
| Addiction | 0 (0.0) | 0 (0.0) | | | | 9 (52.9) | 5 (29.4) | | | | 3 (17.6) | | |
| Psilocybin with psychological support can be a therapeutic tool for those with eating disorders | | | | | | | | | | | | | |
| Anxiety/Depression | 0 (0.0) | 2 (5.4) | | | | 31 (83.8) | 4 (10.8) | | | 0 (0.0) | | | |
| BPAD | 0 (0.0) | 2 (16.7) | | | | 8 (66.7) | 1 (8.3) | | | 1 (8.3) | | | |
| Psychotic | 1 (6.3) | 2 (12.5) | | | | 12 (75) | 1 (6.3) | | | 0 (0.0) | | | |
| PD | 0 (0.0) | 1 (7.1) | | | | 12 (85.7) | 0 (0.0) | | | 1 (7.1) | | | |
| Addiction | 1 (5.9) | 2 (11.8) | | | | 9 (52.9) | 3 (17.6) | | | 2 (11.8) | | | |
| Psilocybin with psychological support can be a therapeutic tool for those with drug or alcohol addiction | | | | | | | | | | | | | |
| Anxiety/Depression | 0 (0.0) | 4 (11.4) | | | | 25 (71.4) | 6 (17.1) | | | | 0 (0.0) | | |
| BPAD | 0 (0.0) | 2 (20.0) | | | | 6 (60.0) | 0 (0.0) | | | | 2 (20.0) | | |
| Psychotic | 3 (20.0) | 2 (13.3) | | | | 8 (53.3) | 1 (6.7) | | | | 1 (6.7) | | |
| PD | 0 (0.0) | 3 (21.4) | | | | 8 (57.1) | 3 (21.4) | | | | 0 (0.0) | | |
| Addiction | 0 (0.0) | 2 (12.5) | | | | 9 (56.3) | 2 (12.5) | | | | 3 (18.8) | | |
| Psilocybin with psychological support can be a therapeutic tool for those with schizophrenia | | | | | | | | | | | | | |
| Anxiety/Depression | 0 (0.0) | 3 (8.1) | | | | 33 (89.2) | 1 (2.7) | | | 0 (0.0) | | | |
| BPAD | 1 (9.1) | 2 (18.2) | | | | 6 (54.5) | 1 (9.1) | | | 1 (9.1) | | | |
| Psychotic | 4 (25.0) | 1 (6.3) | | | | 9 (56.3) | 0 (0.0) | | | 2 (12.5) | | | |
| PD | 1 (7.1) | 2 (14.3) | | | | 8 (57.1) | 2 (14.3) | | | 1 (7.1) | | | |
| Addiction | 0 (0.0) | 2 (11.8) | | | | 10 (58.8) | 4 (23.5) | | | 1 (5.9) | | | |
| **Attitudes to the safety & legal aspect of psilocybin** | | | | | | | | | | | | | |
| Psilocybin should be tested for medicinal value | | | | | | | | | | | | | |
| Anxiety/Depression | 2 (5.4) | 0 (0.0) | | | | 9 (24.3) | 14 (37.8) | | | | 12 (32.4) | | |
| BPAD | 0 (0.0) | 2 (18.2) | | | | 4 (36.4) | 1 (9.1) | | | | 4 (36.4) | | |
| Psychotic | 0 (0.0) | 3 (18.8) | | | | 5 (31.3) | 3 (18.8) | | | | 5 (31.3) | | |
| PD | 0 (0.0) | 0 (0.0) | | | | 0 (0.0) | 5 (35.7) | | | | 9 (64.3) | | |
| Addiction | 0 (0.0) | 0 (0.0) | | | | 1 (5.9) | 5 (29.4) | | | | 11 (64.7) | | |
| Psilocybin should be granted medical treatment status | | | | | | | | | | | | | |
| Anxiety/Depression | 0 (0.0) | 1 (2.7) | | | | 14 (37.8) | 14 (37.8) | | | | 8 (21.6) | | |
| BPAD | 0 (0.0) | 2 (22.2) | | | | 2 (22.2) | 2 (22.2) | | | | 3 (33.3) | | |
| Psychotic | 0 (0.0) | 3 (18.8) | | | | 7 (43.8) | 1 (6.3) | | | | 5 (31.3) | | |
| PD | 0 (0.0) | 1 (7.1) | | | | 3 (21.4) | 4 (28.6) | | | | 6 (42.9) | | |
| Addiction | 0 (0.0) | 0 (0.0) | | | | 6 (35.3) | 6 (35.3) | | | | 5 (29.4) | | |
| The government should fund studies to explore medicinal uses for Psilocybin | | | | | | | | | | | | | |
| Anxiety/Depression | 0 (0.0) | 2 (5.4) | | | | 11 (29.7) | 10 (27.0) | | | | 14 (37.8) | | |
| BPAD | 1 (8.3) | 3 (25.0) | | | | 3 (25.0) | 3 (25.0) | | | | 2 (16.7) | | |
| Psychotic | 0 (0.0) | 2 (12.5) | | | | 5 (31.3) | 4 (25.0) | | | | 5 (31.3) | | |
| PD | 2 (14.3) | 2 (14.3) | | | | 2 (14.3) | 3 (21.4) | | | | 5 (35.7) | | |
| Addiction | 2 (11.8) | 5 (29.4) | | | | 7 (41.2) | 2 (11.8) | | | | 1 (5.9) | | |
| Psilocybin can be safely enjoyed when used recreationally | | | | | | | | | | | | | |
| Anxiety/Depression | 5 (13.5) | 2 (5.4) | | | | 18 (48.6) | 9 (24.3) | | | | 3 (8.1) | | |
| BPAD | 1 (9.1) | 4 (36.4) | | | | 2 (18.2) | 2 (18.2) | | | | 2 (18.2) | | |
| Psychotic | 3 (18.8) | 3 (18.8) | | | | 8 (50.0) | 2 (12.5) | | | | 0 (0.0) | | |
| PD | 1 (7.1) | 2 (14.3) | | | | 3 (21.4) | 5 (35.7) | | | | 3 (21.4) | | |
| Addiction | 1 (6.3) | 2 (12.5) | | | | 5 (31.3) | 8 (50.0) | | | | 0 (0.0) | | |
| Psychedelics should be illegal for recreational purposes | | | | | | | | | | | | | |
| Anxiety/Depression | 0 (0.0) | 13 (35.1) | | | | 11 (29.7) | 10 (27.0) | | | | 3 (8.1) | | |
| BPAD | 3 (25.0) | 1 (8.3) | | | | 2 (16.7) | 6 (50.0) | | | | 0 (0.0) | | |
| Psychotic | 1 (6.3) | 3 (18.8) | | | | 6 (37.5) | 4 (25.0) | | | | 2 (12.5) | | |
| PD | 4 (28.6) | 4 (28.6) | | | | 4 (28.6) | 1 (7.1) | | | | 1 (7.1) | | |
| Addiction | 3 (17.6) | 5 (29.4) | | | | 7 (41.2) | 2 (11.8) | | | | 0 (0.0) | | |
| Psychedelics are unsafe even under medical supervision | | | | | | | | | | | | | |
| Anxiety/Depression | 4 (10.8) | 11 (29.7) | | | | 20 (54.1) | 2 (5.4) | | | | 0 (0.0) | | |
| BPAD | 2 (18.2) | 2 (18.2) | | | | 3 (27.3) | 4 (36.4) | | | | 0 (0.0) | | |
| Psychotic | 1 (5.9) | 2 (11.8) | | | | 4 (47.1) | 4 (23.5) | | | | 2 (11.8) | | |
| PD | 2 (14.3) | 7 (50.0) | | | | 2 (14.3) | 1 (7.1) | | | | 2 (14.3) | | |
| Addiction | 2 (11.8) | 6 (35.3) | | | | 5 (29.4) | 2 (11.8) | | | | 2 (11.8) | | |
| **Attitudes and Acceptability** | | | | | | | | | | | | | |
| Psilocybin with psychological support would be useful for my mental health problem | | | | | | | | | | | | | |
| Anxiety/Depression | 1 (2.7) | | 5 (13.5) | | | 21 (56.8) | | | 8 (21.6) | | | 2 (5.4) | |
| BPAD | 0 (0.0) | | 0 (0.0) | | | 10 (63.3) | | | 1 (8.3) | | | 1 (8.3) | |
| Psychotic | 3 (18.8) | | 3 (18.8) | | | 7 (43.8) | | | 1 (6.3) | | | 2 (12.5) | |
| PD | 1 (7.1) | | 2 (14.3) | | | 6 (42.9) | | | 2 (14.3) | | | 3 (21.4) | |
| Addiction | 1 (5.9) | | 2 (8.11) | | | 5 (29.4) | | | 2 (11.8) | | | 1 (41.2) | |
| I would accept Psilocybin with psychological support if a doctor recommended it | | | | | | | | | | | | | |
| Anxiety/Depression | 2 (5.4) | | | | 4 (10.8) | 11 (29.7) | | 14 (37.8) | | | | | 6 (16.2) |
| BPAD | 0 (0.0) | | | | 3 (25.0) | 3 (25.0) | | 4 (33.3) | | | | | 2 (16.7) |
| Psychotic | 3 (18.8) | | | | 3 (18.8) | 6 (37.5) | | 2 (12.5) | | | | | 2 (12.5) |
| PD | 2 (14.3) | | | | 2 (14.3) | 0 (0.0) | | 5 (35.7) | | | | | 5 (35.7) |
| Addiction | 0 (0.0) | | | | 1 (5.9) | 3 (17.6) | | 6 (35.3) | | | | | 7 (41.2) |
| I would be willing to come off my medications to accept Psilocybin with psychological support | | | | | | | | | | | | | |
| Anxiety/Depression | 1 (2.7) | | | 6 (16.2) | | 4 (10.8) | | 14 (37.8) | | | | | 5 (13.5) |
| BPAD | 5 (41.7) | | | 0 (0.0) | | 2 (16.7) | | 3 (25.0) | | | | | 2 (16.7) |
| Psychotic | 3 (17.6) | | | 5 (29.4) | | 4 (23.5) | | 4 (23.5) | | | | | 1 (5.9) |
| PD | 2 (14.3) | | | 2 (14.3) | | 1 (7.1) | | 5 (35.7) | | | | | 4 (28.6) |
| Addiction | 0 (0.0) | | | 2 (11.8) | | 2 (11.8) | | 4 (23.5) | | | | | 7 (41.2) |
| I would say I am knowledgeable about psychedelic drugs | | | | | | | | | | | | | |
| Anxiety/Depression | 12 (33.3) | | | 8 (22.2) | | 9 (25.0) | | 6 (16.7) | | | | | 1 (2.8) |
| BPAD | 3 (27.3) | | | 3 (27.3) | | 1 (9.1) | | 2 (18.2) | | | | | 2 (18.2) |
| Psychotic | 2 (13.3) | | | 1 (6.7) | | 4 (26.7) | | 6 (40.0) | | | | | 2 (13.3) |
| PD | 1 (7.1) | | | 3 (21.4) | | 4 (28.6) | | 6 (42.9) | | | | | 0 (0.0) |
| Addiction | 1 (5.9) | | | 8 (47.1) | | 1 (5.9) | | 6 (35.3) | | | | | 1 (5.9) |
| I would say I am knowledgeable about magic mushrooms | | | | | | | | | | | | | |
| Anxiety/Depression | 14 (38.9) | | | 9 (25.0) | | 8 (22.2) | | 5 (13.9) | | | | | 0 (0.0) |
| BPAD | 2 (16.7) | | | 4 (33.3) | | 2 (16.7) | | 3 (25.0) | | | | | 1 (8.3) |
| Psychotic | 3 (20.0) | | | 4 (26.7) | | 2 (13.3) | | 5 (33.3) | | | | | 1 (6.7) |
| PD | 1 (7.7) | | | 5 (38.5) | | 3 (23.1) | | 3 (23.1) | | | | | 1 (7.1) |
| Addiction | 1 (5.9) | | | 8 (47.1) | | 3 (17.6) | | 3 (17.6) | | | | | 2 (11.8) |
| Psilocybin can increase people’s connection to nature | | | | | | | | | | | | | |
| Anxiety/Depression | 1 (2.7) | | | 0 (0.0) | | 24 (64.9) | | 8 (21.6) | | | | | 4 (10.8) |
| BPAD | 0 (0.0) | | | 3 (25.3) | | 5 (41.7) | | 1 (8.3) | | | | | 3 (25.0) |
| Psychotic | 1 (6.3) | | | 4 (25.0) | | 8 (50.0) | | 1 (6.3) | | | | | 2 (12.5) |
| PD | 0 (0.0) | | | 1 (7.1) | | 6 (42.9) | | 4 (28.6) | | | | | 3 (21.4) |
| Addiction | 0 (0.0) | | | 0 (0.0) | | 4 (23.5) | | 6 (35.3) | | | | | 7 (41.2) |
| Psilocybin can increase people’s connection to other people | | | | | | | | | | | | | |
| Anxiety/Depression | 0 (0.0) | | | 2 (5.4) | | 23 (62.2) | | 10 (27.0) | | | | | 2 (5.4) |
| BPAD | 0 (0.0) | | | 2 (16.7) | | 6 (50.0) | | 2 (16.7) | | | | | 2 (16.7) |
| Psychotic | 0 (0.0) | | | 5 (31.3) | | 8 (50.0) | | 1 (6.3) | | | | | 2 (12.5) |
| PD | 1 (7.1) | | | 0 (0.0) | | 8 (57.1) | | 4 (28.6) | | | | | 1 (7.1) |
| Addiction | 0 (0.) | | | 4 (23.5) | | 4 (23.5) | | 4 (23.5) | | | | | 5 (29.4) |
| Psilocybin can lead to a mystical experience | | | | | | | | | | | | | |
| Anxiety/Depression | 0 (0.0) | | | 1 (2.7) | | 22 (59.5) | | 13 (35.1) | | | | | 1 (2.7) |
| BPAD | 1 (8.3) | | | 0 (0.0) | | 5 (41.7) | | 5 (41.7) | | | | | 1 (8.3) |
| Psychotic | 0 (0.0) | | | 0 (0.0) | | 9 (56.3) | | 4 (25.0) | | | | | 3 (18.6) |
| PD | 0 (0.0) | | | 0 (0.0) | | 4 (28.6) | | 6 (42.3) | | | | | 4 (28.6) |
| Addiction | 0 (0.0) | | | 1 (5.9) | | 4 (23.5) | | 8 (47.1) | | | | | 4 (23.5) |
| Psilocybin can be addictive | | | | | | | | | | | | | |
| Anxiety/Depression | 0 (0.0) | | | 7 (19.4) | | 24 (66.7) | | 4 (11.1) | | | | | 1 (2.8) |
| BPAD | 1 (9.1) | | | 3 (27.3) | | 4 (36.4) | | 3 (27.3) | | | | | 0 (0.0) |
| Psychotic | 2 (12.5) | | | 2 (12.5) | | 8 (50.0) | | 3 (18.8) | | | | | 1 (6.3) |
| PD | 1 (7.1) | | | 4 (28.6) | | 3 (21.4) | | 6 (42.9) | | | | | 0 (0.0) |
| Addiction | 3 (17.6) | | | 7 (41.2) | | 5 (29.4) | | 1 (5.9) | | | | | 1 (5.9) |

**Table S9: Attitudes by diagnosis** (net agree)

| **Diagnosis** | **Depression/**  **Anxiety**  count (% within group agree excluding neutral) | **Bipolar** | **Psychotic disorders** | **Personality disorders** | **Addiction** | Pearson Chi-squared p value |
| --- | --- | --- | --- | --- | --- | --- |
| Previous psychedelic use | 7 (20) | 5 (14.3) | 3 (8.6) | 8 (22.9) | 12 (34.3) | *.002*** |
| Knowledgeable about psychedelics | 7 (21.9) | 4 (12.5) | 8 (25.0) | 6 (18.8) | 7 (21.9) | .078 |
| Knowledgeable about magic mushrooms | 5 (20.8) | 4 (16.7) | 6 (25.0) | 4 (16.7) | 5 (20.8) | .349 |
| Psilocybin for some mental health disorders | 12 (35.3) | 4 (11.8) | 4 (11.8) | 5 (14.7) | 9 (26.5) | .06 |
| Psilocybin for depression | 10 (34.5) | 4 (13.8) | 2 (6.9) | 5 (17.2) | 8 (27.6) | .036 |
| Psilocybin for anxiety | 7 (31.8) | 3 (13.6) | 2 (9.1) | 3 (13.6) | 7 (31.8) | .372 |
| Psilocybin addictive | 5 (25) | 3 (15) | 4 (20) | 6 (30) | 2 (10) | .398 |
| Psilocybin safe recreationally | 12 (35.3) | 4 (11.8) | 2 (5.9) | 8 (23.5) | 8 (23.5) | .169 |
| Psilocybin for smoking cessation | 2 (18.2) | 2 (18.2) | 1 (9.1) | 1 (9.1) | 5 (45.5) | .675 |
| Psilocybin for psychotic disorders | 1 (7.7) | 2 (15.4) | 2 (15.4) | 3 (23.1) | 5 (38.5) | .478 |
| Psilocybin for drug and alcohol addiction | 6 (33.3) | 2 (11.1) | 2 (11.1) | 3 (16.7) | 5 (27.8) | .581 |
| Psilocybin for chronic pain | 9 (28.1) | 3 (9.4) | 6 (18.8) | 6 (18.8) | 8 (25) | .110 |
| Psilocybin for eating disorders | 4 (30.8) | 2 (15.4) | 1 (7.7) | 1 (7.7) | 5 (38.5) | .733 |
| Psilocybin increases connection to nature | 12 (30.8) | 4 (10.3) | 3 (7.7) | 7 (17.9) | 13 (33.3) | *.003*** |
| Psilocybin increases connection to others | 12 (36.4) | 4 (12.1) | 3 (9.1) | 5 (15.2) | 9 (27.3) | .182 |
| Psilocybin can lead to mystical experiences | 14 (28.9) | 6 (12.2) | 7 (14.3) | 10 (20.4) | 12 (24.5) | .720 |
| Psychedelics unsafe even under medical supervision | 2 (10.5) | 4 (21.1) | 6 (31.6) | 3 (15.8) | 4 (21.1) | *.050** |
| Psilocybin should be tested for medicinal value | 26 (37.7) | 5 (7.2) | 8 (11.6) | 14 (20.3) | 16 (23.2) | *.031** |
| Psilocybin illegal for recreational use | 13 (44.8) | 6 (20.7) | 6 (20.7) | 2 (6.9) | 2 (6.9) | .129 |
| Government should fund psilocybin studies | 24 (49) | 5 (10.2) | 9 (18.4) | 8 (16.3) | 3 (6.1) | *.003*** |
| Psilocybin granted medical treatment status | 22 (40.7) | 5 (9.3) | 6 (11.1) | 10 (18.5) | 11 (20.4) | .064 |
| Psilocybin would benefit my condition | 10 (34.5) | 2 (6.9) | 3 (10.3) | 5 (17.2) | 9 (31) | .266 |
| Would accept psilocybin if doctor recommended | 20 (37.7) | 6 (11.3) | 4 (7.5) | 10 (18.9) | 13 (24.5) | .071 |
| Willing to come off medications | 19 (38.8) | 5 (10.2) | 5 (10.2) | 9 (18.4) | 11 (22.4) | .088 |

**Table S10: Attitudes by possible indication vs contra-indicated diagnoses**

|  | **Possible indication**  **agree vs disagree** (excluding neutral)  count (within group %) | | **Contra-indicated** | | Pearson Chi-squared p value |
| --- | --- | --- | --- | --- | --- |
|  | No/disagree | Yes/agree | No/disagree | Yes/agree |  |
| Previous psychedelic use | 36 (65.5) | 19 (34.5) | 21 (72.4) | 8 (27.6) | 0.51 |
| Knowledgeable about psychedelics | 29 (67.4) | 14 (32.6) | 9 (42.9) | 12 (57.1) | 0.06 |
| Knowledgeable about magic mushrooms | 32 (76.2) | 10 (23.8) | 13 (56.5) | 10 (43.5) | 0.10 |
| Psilocybin for some mental health disorders | 3 (12.5) | 21 (87.5) | 10 (55.6) | 8 (44.4) | *0.003*** |
| Psilocybin for depression | 2 (10) | 18 (90) | 8 (57.1) | 6 (42.9) | *0.003*** |
| Psilocybin for anxiety | 6 (30) | 14 (70) | 9 (64.3) | 5 (35.7) | *0.048** |
| Psilocybin addictive | 17 (78.8) | 7 (22.9) | 8 (53.3) | 7 (47.7) | 0.26 |
| Psilocybin safe recreationally | 10 (33.3) | 20 (66.6) | 11 (64.7) | 6 (35.3) | *0.038** |
| Psilocybin for smoking cessation | 7 (50) | 7 (50) | 8 (72.7) | 3 (27.3) | 0.25 |
| Psilocybin for psychotic disorders | 5 (45.5) | 6 (54.5) | 8 (66.7) | 4 (33.3) | 0.30 |
| Psilocybin for drug and alcohol addiction | 6 (35.3) | 11 (64.7) | 7 (63.6) | 4 (36.4) | 0.14 |
| Psilocybin for chronic pain | 1 (5.6) | 17 (94.4) | 5 (35.7) | 9 (64.3) | 0.03* |
| Psilocybin for eating disorders | 5 (35.7) | 9 (64.3) | 5 (62.5) | 3 (37.5) | 0.22 |
| Psilocybin increases connection to nature | 1 (3.8) | 25 (96.2) | 8 (53.3) | 7 (46.7) | *0.0002**** |
| Psilocybin increases connection to others | 6 (22.2) | 21 (77.8) | 7 (50) | 7 (50) | 0.07 |
| Psilocybin can lead to mystical experiences | 2 (7.1) | 26 (92.9) | 1 (7.1) | 13 (92.9) | 1.0 |
| Psychedelics unsafe even under medical supervision | 23 (79.3) | 6 (20.7) | 7 (41.2) | 10 (58.8) | *0.009*** |
| Psilocybin should be tested for medicinal value | 2 (4.5) | 42 (95.5) | 5 (27.8) | 13 (72.2) | *0.009*** |
| Psilocybin should be illegal for recreational use | 21 (58.3) | 15 (41.7) | 8 (40) | 12 (60) | 0.18 |
| Government should fund psilocybin studies | 9 (25) | 27 (75) | 6 (30) | 14 (70) | 0.68 |
| Psilocybin granted medical treatment status | 1 (2.9) | 33 (97.1) | 5 (31.3) | 11 (68.8) | *0.004*** |
| Psilocybin would benefit my condition | 9 (32.1) | 19 (67.9) | 6 (54.5) | 5 (45.5) | 0.19 |
| Would accept psilocybin if doctor recommended | 7 (17.5) | 33 (82.5) | 9 (47.4) | 10 (52.6) | *0.016** |
| Willing to come off medications | 9 (23.1) | 33 (76.9) | 13 (56.5) | 10 (43.5) | *0.008** |

**Table S11: Attitudes by Education**

|  | **Secondary School**  count (% within group) | | **University**  Count (% within group) | | Pearson Chi-squared p value |
| --- | --- | --- | --- | --- | --- |
|  | No/disagree | Yes/agree | No/disagree | Yes/agree |  |
| Previous psychedelic use | 35 (64.8) | 19 (35.2) | 29 (64.4) | 16 (35.6) | .969 |
| Knowledgeable about psychedelics | 24 (58.5) | 17 (41.5) | 18 (52.9) | 16 (47.1) | .627 |
| Knowledgeable about magic mushrooms | 28 (71.8) | 11 (28.2) | 24 (64.9) | 13 (35.1) | .516 |
| Psilocybin for some mental health disorders | 9 (31.0) | 20 (69.0) | 6 (30) | 14 (70) | .938 |
| Psilocybin for depression | 6 (25.0) | 18 (75) | 6 (35.3) | 11 (64.7) | .475 |
| Psilocybin for anxiety | 8 (40) | 12 (60) | 9 (45) | 11 (55) | .749 |
| Psilocybin addictive | 14 (51.9) | 13 (48.1) | 17 (70.8) | 7 (29.2) | .166 |
| Psilocybin safe recreationally | 11 (39.3) | 17 (60.7) | 13 (43.3) | 17 (56.7) | .754 |
| Psilocybin for smoking cessation | 10 (58.8) | 7 (41.2) | 7 (63.6) | 4 (36.4) | .799 |
| Psilocybin for psychotic disorders | 7 (43.8) | 9 (56.3) | 9 (64.3) | 5 (35.7) | .261 |
| Psilocybin for drug and alcohol addiction | 12 (54.5) | 10 (45.5) | 5 (38.5) | 8 (61.5) | .358 |
| Psilocybin for chronic pain | 4 (17.4) | 19 (82.6) | 2 (12.5) | 14 (87.5) | .677 |
| Psilocybin for eating disorders | 6 (40) | 9 (60) | 5 (55.6) | 4 (44.4) | .459 |
| Psilocybin increases connection to nature | 5 (17.9) | 23 (82.1) | 5 (23.8) | 16 (76.2) | .609 |
| Psilocybin increases connection to others | 7 (25.9) | 20 (74.1) | 8 (38.1) | 13 (61.9) | .367 |
| Psilocybin can lead to mystical experiences | 1 (3.8) | 25 (96.2) | 2 (7.4) | 25 (92.6) | .575 |
| Psychedelics unsafe even under medical supervision | 20 (64.5) | 11 (35.5) | 20 (71.4) | 8 (28.6) | .570 |
| Psilocybin should be tested for medicinal value | 4 (9.3) | 39 (90.7) | 3 (9.1) | 30 (90.3) | .975 |
| Psilocybin illegal recreational use | 18 (51.4) | 17 (48.6) | 19 (61.3) | 12 (38.7) | .420 |
| Government should fund psilocybin studies | 8 (21.6) | 29 (78.4) | 11 (34.4) | 21 (65.6) | .237 |
| Psilocybin granted medical treatment status | 4 (11.1) | 32 (88.9) | 3 (11.5) | 23 (88.5) | .958 |
| Psilocybin would benefit my condition | 8 (33.3) | 16 (66.7) | 10 (43.5) | 13 (56.5) | .474 |
| Would accept psilocybin if doctor recommended | 11 (26.8) | 30 (73.2) | 9 (28.1) | 23 (71.9) | .902 |
| Willing to come off medications | 14 (35) | 26 (65) | 13 (36.1) | 23 (63.9) | .920 |

**Table S12: Attitudes by Employment** (excluding retired/student)

| **Employment** | **Unemployed**  count (% within group) | | **Employed - Full and Part Time**  count (% within group) | | Pearson Chi-squared p value |
| --- | --- | --- | --- | --- | --- |
|  | No/disagree | Yes/agree | No/disagree | Yes/agree |  |
| Previous psychedelic use | 19 (61.3) | 12 (38.7) | 28 (58.3) | 20 (41.7) | .794 |
| Knowledgeable about psychedelics | 11 (47.8) | 12 (52.2) | 19 (52.8) | 17 (47.2) | .711 |
| Knowledgeable about magic mushrooms | 15 (68.2) | 7 (31.8) | 22 (57.9) | 16 (42.1) | .430 |
| Psilocybin for some mental health disorders | 6 (31.6) | 13 (68.4) | 3 (13) | 20 (87) | .145 |
| Psilocybin for depression | 2 (18.2) | 9 (81.8) | 4 (18.2) | 18 (81.8) | 1.0 |
| Psilocybin for anxiety | 4 (36.4) | 7 (63.6) | 6 (27.3) | 16 (72.7) | .592 |
| Psilocybin addictive | 11 (61.1) | 7 (38.9) | 18 (66.7) | 9 (33.3) | .703 |
| Psilocybin safe recreationally | 7 (38.9) | 11 (61.1) | 11 (34.4) | 21 (64.0) | .750 |
| Psilocybin for smoking cessation | 7 (70) | 3 (30) | 6 (42.9) | 8 (57.1) | .189 |
| Psilocybin for psychotic disorders | 5 (50) | 5 (50) | 8 (50) | 8 (50) | 1.0 |
| Psilocybin for drug and alcohol addiction | 7 (70) | 3 (30) | 4 (21.1) | 15 (78.9) | *.010** |
| Psilocybin for chronic pain | 3 (20) | 12 (80) | 1 (5.9) | 16 (94.1) | .228 |
| Psilocybin for eating disorders | 3 (37.5) | 5 (62.5) | 5 (38.5) | 8 (61.5) | .965 |
| Psilocybin increases connection to nature | 5 (27.8) | 13 (72.2) | 3 (11.5) | 23 (88.5) | .170 |
| Psilocybin increases connection to others | 4 (23.5) | 13 (76.5) | 8 (29.6) | 19 (70) | .658 |
| Psilocybin can lead to mystical experiences | 1 (5.9) | 16 (94.1) | 2 (6.7) | 28 (93.3) | .916 |
| Psychedelics unsafe even under medical supervision | 11 (64.7) | 6 (35.3) | 24 (72.7) | 9 (27.3) | .558 |
| Psilocybin should be tested for medicinal value | 2 (8.3) | 22 (91.7) | 2 (5.4) | 35 (94.6) | .652 |
| Psilocybin illegal recreationally | 10 (47.6) | 11 (52.4) | 23 (71.9) | 9 (28.1) | .075 |
| Government should fund psilocybin studies | 6 (25) | 18 (75) | 12 (33.3) | 24 (66.7) | .490 |
| Psilocybin granted medical treatment status | 3 (14.3) | 18 (85.7) | 2 (6.3) | 30 (93.8) | .328 |
| Psilocybin would benefit my condition | 6 (40) | 9 (60) | 7 (26.9) | 19 (73.1) | .386 |
| Would accept psilocybin if doctor recommended | 9 (37.5) | 15 (62.5) | 6 (15.8) | 32 (84.2) | .052 |
| Willing to come off medications | 9 (37.5) | 15 (62.5) | 11 (28.2) | 28 (71.8) | .441 |

**Questionnaire**

| **Participant ID Number** | | | **Date:** | | |
| --- | --- | --- | --- | --- | --- |
| **Age** |  | | | | |
| **Nationality** |  | | | | |
| **Gender** | | | | | |
| **Male** |  | | | | |
| **Female** |  | | | | |
| **Other** |  | | | | |
| **Education** | | | | | |
| **Left school before age 16 without qualifications** |  | | | | |
| **Some high school/Junior Cert level** |  | | | | |
| **High school diploma/Leaving Cert level** |  | | | | |
| **Some university** (or equivalent) |  | | | | |
| **Bachelor’s degree** (or equivalent) |  | | | | |
| **Post-graduate degree** (e.g., Masters or Doctorate) |  | | | | |
| **Employment** | | | | | |
| **Student** |  | | | | |
| **Unemployed** |  | | | | |
| **Part-time job** |  | | | | |
| **Full-time job** |  | | | | |
| **Retired** |  | | | | |
| **Religion** | | | | | |
| **None** |  | | | | |
| **Christian** (all denominations) |  | | | | |
| **Muslim** |  | | | | |
| **Buddhist** |  | | | | |
| **Hindu** |  | | | | |
| **Jewish** |  | | | | |
| **Sikh** |  | | | | |
| **Other** | | | | | |
| **What category would best describe my diagnosis** (single best fit) | | | | | |
| **Depression** |  | | | | |
| **Bipolar Disorder** |  | | | | |
| **Psychotic disorder** (Schizophrenia, schizoaffective disorder, Delusional disorder) |  | | | | |
| **Generalized Anxiety disorder** |  | | | | |
| **Post-traumatic Stress Disorder** |  | | | | |
| **Panic Disorder** |  | | | | |
| **Obsessive Compulsive disorder** |  | | | | |
| **Eating disorder** |  | | | | |
| **Emotionally unstable personality disorder** (Borderline personality disorder) |  | | | | |
| **Other personality disorder** |  | | | | |
| **Alcohol addiction** |  | | | | |
| **Drug addiction** (please specify) |  | | | | |
| **Other diagnosis** (please specify) |  | | | | |
| **Previous psychedelic drug use**  (Psilocybin (magic mushrooms), LSD, DMT, Mescaline/Peyote) | | | | | |
| **Never** (psychedelic naïve) |  | | | | |
| **Once** |  | | | | |
| **2–5 times** |  | | | | |
| **6–20 times** |  | | | | |
| **More than 21 times** |  | | | | |
| **Have you ever attended a psychedelic retreat?** | | | | | |
| **Yes** |  | | | | |
| **No** |  | | | | |
| **If you answered yes to the above question;** | | | | | |
| **Were you asked about any history of mental illness?** | **Yes** |  | | **No** |  |
| **Were you asked what medications you were taking?** | **Yes** |  | | **No** |  |

| **Have you ever used any of the following drugs, even if just once?** | | | | | | |
| --- | --- | --- | --- | --- | --- | --- |
| **Substance groups** | **Specific substances** | **EVER used drug** | **Used drug(s) during the last 12 MONTHS** | **Used drug(s) during the last 30 DAYS** | **Age at first use** | **Age at last use** |
| **Psychedelics** | **Psilocybin (magic mushrooms)** |  |  |  |  |  |
|  | **LSD** |  |  |  |  |  |
|  | **DMT** |  |  |  |  |  |
|  | **Mescaline/Peyote** |  |  |  |  |  |
|  | **Salvia** |  |  |  |  |  |
|  | **Microdosing** |  |  |  |  |  |
|  | If Microdosing  (specify substance) |  | | | | |
| **Ketamine** |  |  |  |  |  |  |
| **Tobacco** |  |  |  |  |  |  |
| **Alcohol** |  |  |  |  |  |  |
| **Cannabis** |  |  |  |  |  |  |
| **Cocaine** | **Powder cocaine** |  |  |  |  |  |
|  | **Crack** |  |  |  |  |  |
| **Stimulants** | **Ecstasy** |  |  |  |  |  |
|  | **MDMA** |  |  |  |  |  |
|  | **Amphetamines** |  |  |  |  |  |
|  | **Methamphetamines** |  |  |  |  |  |
|  | **Others** |  |  |  |  |  |
| **Hypnotics/Sedatives (non-prescribed)** | **Barbiturates misused** |  |  |  |  |  |
|  | **Benzodiazepines misused** |  |  |  |  |  |
|  | **GHB/GBL** |  |  |  |  |  |
|  | **Other hypnotics/sedatives** |  |  |  |  |  |
| **Volatile inhalants/solvents** |  |  |  |  |  |  |
| **Opioids** |  |  |  |  |  |  |
| **Anabolic steroids** |  |  |  |  |  |  |
| **Other substances** (specify) |  |  |  |  |  |  |

**Please answer the following questions by circling the most appropriate answer**

**My experiences with psychedelic drugs were positive**

Strongly Disagree Disagree Neutral Agree Strongly Agree

Not applicable

**I would say I am knowledgeable about psychedelic drugs**

Strongly Disagree Disagree Neutral Agree Strongly Agree

**I would say I am knowledgeable about Psilocybin (magic mushrooms)**

Strongly Disagree Disagree Neutral Agree Strongly Agree

**Psilocybin (magic mushrooms) if used with psychological support shows promise in the treatment of some mental health problems**

Strongly Disagree Disagree Neutral Agree Strongly Agree

**Psilocybin (magic mushrooms) with psychological support can be a therapeutic tool for those with depression**

Strongly Disagree Disagree Don’t Know Agree Strongly Agree

**Psilocybin (magic mushrooms) with psychological support can be a therapeutic tool for those with anxiety**

Strongly Disagree Disagree Don’t Know Agree Strongly Agree

**Psilocybin (magic mushrooms) can be addictive**

Strongly Disagree Disagree Don’t Know Agree Strongly Agree

**Psilocybin (magic mushrooms) can be safely enjoyed when used recreationally, much like alcohol or tobacco**

Strongly Disagree Disagree Don’t Know Agree Strongly Agree

**Psilocybin (magic mushrooms) with psychological support can be a therapeutic tool to aid in smoking cessation**

Strongly Disagree Disagree Don’t Know Agree Strongly Agree

**Psilocybin (magic mushrooms) with psychological support can be a therapeutic tool for those with psychotic disorders such as schizophrenia**

Strongly Disagree Disagree Don’t Know Agree Strongly Agree

**Psychedelic drugs are unsafe even under medical/psychiatric supervision**

Strongly Disagree Disagree Don’t Know Agree Strongly Agree

**Psilocybin (magic mushrooms) should be tested for their medicinal value**

Strongly Disagree Disagree Neutral Agree Strongly Agree

**Psilocybin (magic mushrooms) with psychological support can be a therapeutic tool for those with drug or alcohol addiction**

Strongly Disagree Disagree Don’t Know Agree Strongly Agree

**Ps****ilocybin (magic mushrooms) with psychological support can be a therapeutic tool for those with chronic pain**

Strongly Disagree Disagree Don’t Know Agree Strongly Agree

**Psilocybin (magic mushrooms) with psychological support can be a therapeutic tool for those with eating disorders**

Strongly Disagree Disagree Don’t Know Agree Strongly Agree

**Psilocybin (magic mushrooms) can increase people’s connection to nature**

Strongly Disagree Disagree Don’t Know Agree Strongly Agree

**Psilocybin (magic mushrooms) can increase people’s connection to other people**

Strongly Disagree Disagree Don’t Know Agree Strongly Agree

**Psilocybin (magic mushrooms) can lead to a mystical experience**

Strongly Disagree Disagree Don’t Know Agree Strongly Agree

**Psychedelic drugs should be illegal for recreational purposes**

Strongly Disagree Disagree Don’t Know Agree Strongly Agree

**The government should fund studies to explore medicinal uses for Psilocybin (magic mushrooms)**

Strongly Disagree Disagree Neutral Agree Strongly Agree

**Psilocybin (magic mushrooms) should be granted medical treatment status to be used in licensed facilities under the supervision and care of medical practitioners**

Strongly Disagree Disagree Don’t know Agree Strongly Agree

**Psilocybin (magic mushrooms) with psychological support would be therapeutically useful for my mental health problem**

Strongly Disagree Disagree Don’t know Agree Strongly Agree

**I would accept Psilocybin (magic mushrooms) with psychological support if a doctor recommended it**

Strongly Disagree Disagree Neutral Agree Strongly Agree

**I would be willing to gradually come off my medications in order to accept Psilocybin (magic mushrooms) with psychological support if a doctor recommended it**

Strongly Disagree Disagree Neutral Agree Strongly Agree

Not applicable (I wouldn’t accept it)

**If you wouldn’t accept it, why not?...**
